# A prognostic long non-coding RNA-associated competing endogenous RNA network in head and neck squamous cell carcinoma

**DOI:** 10.7717/peerj.9701

**Published:** 2020-09-15

**Authors:** Chengyao Zhang, Wei Cao, Jiawu Wang, Jiannan Liu, Jialiang Liu, Hao Wu, Siyi Li, Chenping Zhang

**Affiliations:** 1 Department of Oral Maxillofacial-Head and Neck Oncology, Shanghai Ninth People’s Hospital, College of Stomatology, Shanghai Jiao Tong University School of Medicine, Shanghai, Shanghai, China; 2 National Clinical Research Center for Oral Diseases, Shanghai, Shanghai, China; 3 Shanghai Key Laboratory of Stomatology and Shanghai Research Institute of Stomatology, Shanghai, Shanghai, China; 4 Department of Head and Neck Cancer Center, Chongqing University Cancer Hospital & Chongqing Cancer Institute & Chongqing Cancer Hospital, Chongqing, Chongqing, China; 5 Department of Urology, The Second Affiliated Hospital of Chongqing Medical University, Chongqing, Chongqing, China; 6 College of Stomatology, Weifang Medical University, Weifang, Shandong, China; 7 Department of Oral and Maxillofacial-Head and Neck Oncology, Fengcheng Hospital & Shanghai Ninth People’s Hospital (Fengcheng Branch Hospital), College of Stomatology, Shanghai Jiao Tong University School of Medicine, Shanghai, Shanghai, China

**Keywords:** Competing endogenous RNA/ceRNA, Head and neck squamous cell carcinoma/HNSCC, LncRNA, TCGA, Prognosis

## Abstract

**Background:**

This study aimed to develop multi-RNA-based models using a competing endogenous RNA (ceRNA) regulatory network to provide survival risk prediction in head and neck squamous cell carcinoma (HNSCC).

**Methods:**

All long non-coding RNA (lncRNA), microRNA (miRNA), and mRNA expression data and clinicopathological features related to HNSCC were derived from The Cancer Genome Atlas. Differentially expressed RNAs were calculated using R. Prognostic factors were identified using univariate Cox regression analysis. Functional analysis was performed using GO, KEGG pathways, and PPI network. Based on the results, we derived a risk signature and compared high- and low-risk subgroups using LASSO regression analysis. Survival analysis and the relationship between risk signature and clinicopathological features were performed using log-rank tests and Cox regression analysis. A ceRNA regulatory network was constructed, and prognostic lncRNAs and miRNA expression levels were validated in vitro and in vivo.

**Results:**

A list of 207 lncRNAs, 18 miRNAs and 362 mRNAs related to overall survival was established. Five lncRNAs (HOTTIP, LINC00460, RMST, SFTA1P, and TM4SF19-AS1), one miRNA (hsa-miR-206), and one mRNA (STC2) were used to construct the ceRNA network. Three prognostic models contained 13 lncRNAs, eight miRNAs, and 17 mRNAs, which correlated with the patient status, disease-free survival (DFS), stage, grade, T stage, N stage, TP53 mutation status, angiolymphatic invasion, HPV status, and extracapsular spread. KEGG pathway analysis revealed significant enrichment of “Transcriptional misregulation in cancer” and “Neuroactive ligand-receptor interaction.” In addition, HOTTIP, LINC00460, miR-206 and STC2 were validated in GTEx data, GEO microarrays and six HNSCC cell lines.

**Conclusions:**

Our findings clarify the interaction of ceRNA regulatory networks and crucial clinicopathological features. These results show that prognostic biomarkers can be identified by constructing multi-RNA-based prognostic models, which can be used for survival risk prediction in patients with HNSCC.

## Introduction

Head and neck squamous cell carcinoma (HNSCC) has the ninth highest global incidence of all malignancies, and accounted for approximately 53,260 new cases and approximately 10,750 deaths in 2019 in the United States of America (Ward et al., 2019; Siegel, Miller & Jemal, 2020; Thrift & Gudenkauf, 2019). Despite advances in surgical procedures, chemo-radiotherapy, and targeted therapy, clinical outcomes have remained unchanged for decades, with the five-year survival rate ranging from 40% to 50% over the past three decades (Braakhuis, Leemans & Visser, 2014). HNSCC is characterized by a remarkably aggressive biological behavior with a high incidence of lymph node and distant metastasis that leads to poor prognosis (Ding et al., 2019; Sun et al., 2017). The 8th edition of American Joint Commission Cancer (AJCC) introduced depth of invasion and extranodal extension as criteria for T and N stages, respectively (Amin et al., 2017). TNM staging are useful for determination of prognosis and development of strategic treatment plans based on the anatomical situation (Asare et al., 2015). However, even patients with the same TNM stage require different therapeutic approaches. Therefore, identification of additional molecular biomarkers is required to improve prognosis by accounting for the biological heterogeneity of HNSCC.

Differential expression of long non-coding RNAs (lncRNAs) might affect the corresponding function and play an important role in cancer pathogenesis. LncRNAs and microRNAs (miRNAs) are important subclasses of competing endogenous RNAs (ceRNAs), which regulate the protein levels of target genes, thus, participating in all aspects of various cellular biological processes (Lin & Gregory, 2015; Schmitt & Chang, 2016). Accumulating evidence suggests that the ceRNA network could be beneficial for prognosis prediction in patients with HNSCC (Xu et al., 2019; Yin et al., 2019; Pan et al., 2019; Fang et al., 2018; Zhao et al., 2018). Zhao et al. (2018) established a ceRNA regulation network related to HNSCC that included eight differently expressed mRNAs (DEmRNAs), 53 differentially expressed lncRNAs (DElncRNAs), and 16 differentially expressed miRNAs (DEmiRNAs). However, that study and others did not construct a prognostic model for HNSCC that assessed the relationship between ceRNA network RNAs and overall survival (OS). In addition, several important characteristics are closely related to the clinicopathological features of HNSCC, such as TP53 mutation status, angiolymphatic invasion (ALI), human papillomavirus (HPV) status, perineural invasion (PNI), and extracapsular spread (ECS). However, no previous studies have, to the best of our knowledge, assessed the relationship between ceRNA network RNAs and the above clinicopathological characteristics.

In the present study, we comprehensively evaluated the prognostic value of differentially expressed RNAs and developed multi-RNA-based models that predicted OS via the risk signature of HNSCC. In addition, we systematically investigated the relationship between prognostic RNAs and HNSCC clinicopathological features.

## Materials & Methods

### Study population

The RNA sequence data of HNSCC were obtained from The Cancer Genome Atlas (TCGA) database using the Data Transfer Tool, and the corresponding clinical information of patients with HNSCC was downloaded. Sequenced information was provided by the Illumina HiSeq miRNAseq and Illumina HiSeq RNA-seq platforms. This study conforms to the publication guidelines of TCGA.

### Differentially expressed RNAs related to HNSCC

**Figure 1:**
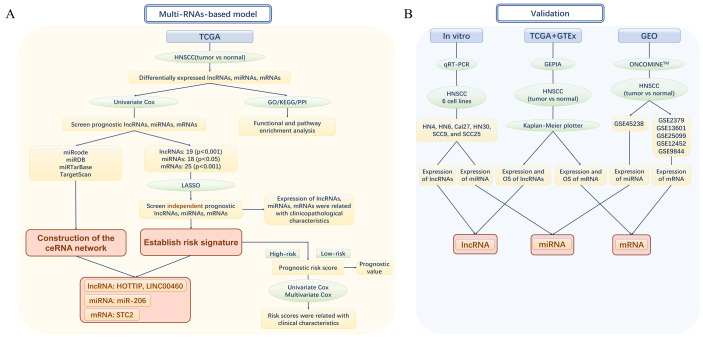
The flow chart of the study design and analysis. (A) The flow chart of the establishment of Multi-RNAs-based models. (B) The flow chart of valifation. TCGA, The Cancer Genome Atlas; HNSCC, Head and neck squamous cell carcinoma; lncRNA, long noncoding RNA; mRNA, messenger RNA; miRNA, microRNA; OS, overall survival.

The design and analytical approach of the present study are shown in the flow chart (Figs. 1A and 1B). lncRNA-seq and mRNA-seq data were obtained from 546 samples consisting of 502 HNSCC and 44 adjacent normal tissues. MiRNA-seq information was obtained from 569 samples consisting of 525 HNSCC and 44 adjacent normal tissue samples. According to the cutoff criteria of —log2 (fold-change [FC]) — > 2 and adjusted *p* < 0.05, DEmRNAs and DEmiRNAs in the HNSCC and normal tissues were identified using the “edgeR” package in R. Next, DElncRNAs were screened out using the same method above in R with the thresholds of —log_2_FC— > 2.0 and adjusted *p*-value < 0.05. Then, the DElncRNAs were annotated and defined using the Encyclopedia of DNA Elements (ENCODE). The statistical significance of multiple comparisons was corrected using FDR (false discovery rate) and FDR < 0.05 was considered statistically significant.

### Survival analysis

Patients with HNSCC with an OS greater than 100 days were included in the survival analysis. Univariate Cox regression analysis was conducted to screen for the prognostic DElncRNAs, DEmRNAs, and DEmiRNAs for OS. Last absolute shrinkage and selection operator (LASSO) regression analysis was conducted with DElncRNAs and DEmRNAs with *p* < 0.001, and DEmiRNAs with *p* < 0.05 for establishment of risk signatures in univariate Cox regression analysis. The risk score (RS) was evaluated using the following formula:

RS = ${\mathop{\sum }\nolimits }_{i=1}^{n}\text{Coef}(i)R(i)$

Where *n* stands for the number of RNAs, Coef (*i*) represents the coefficient, and *R*(*i*) represents the relative gene expression of *z*-score-transformed estimated by LASSO regression analysis. If the RS of a sample was greater than the mean value of the RS, the sample was assigned to the high-risk group; otherwise, it was assigned to the low-risk group. The differences between high- and low-risk patients and OS were evaluated using Kaplan–Meier survival plots and log-rank tests. Moreover, the sensitivity and specificity for survival prediction were estimated by receiver operating characteristic (ROC) curves and areas under the ROC curves (AUC).

### Construction of the ceRNA network

A co-expression network related to prognostic differentially expressed RNAs screened out in univariate Cox regression analysis was established and visualized using Cytoscape software v 3.5.1. The interactions between differentially expressed RNAs were considered as ceRNA triples, which were obtained using the following steps: To obtain the significant co-expressed pairs, DElncRNA and DEmiRNA interaction pairs were retrieved from miRcode (http://www.mircode.org/). Next, DEmiRNA and DEmRNA interaction pairs were predicted based on miRDB (http://www.mirdb.org/), miRTarBase (http://mirtarbase.mbc.nctu.edu.tw/), and TargetScan (http://www.targetscan.org/vert_71/).

### Functional analysis and PPI network

Functional enrichment analyses, including KEGG and GO enrichment analyses, were conducted using the “clusterProfiler” package. The Search Tool for the Retrieval of Interacting Genes (STRING, http://string.embl.de/) was used to build the PPI network according to a combined score greater than 0.4 as the threshold to evaluate the interactive relationships of prognostic DEmRNAs. The nodes with substantially more connections (>5) were considered hub proteins.

### Validation and prognostic values

To test the reliability of the results, the expression levels and prognostic values of the prognostic lncRNAs and mRNAs in the ceRNA network were verified using Gene Expression Profiling Interactive Analysis (GEPIA, http://gepia.cancer-pku.cn/index.html), which was used to analyze the RNA sequencing data from TCGA and Genotype-Tissue Expression (GTEx) dataset projects. The threshold —Log_2_FC— for prognostic factors was determined through boxplot analysis using log_2_—TPM+1—.

The DEmiRNA of miR-206 in the ceRNA network was validated with a microarray from the Gene Expression Omnibus (GEO) database (https://www.ncbi.nlm.nih.gov/geo/) between tumor and normal tissues samples (in vivo), while was validated in cell lines by qRT-PCR (in vitro). Then, the DEmRNA of STC2 in the ceRNA network between tumor and normal tissues samples was validated with six gene expression datasets obtained from ONCOMINE™  (https://www.oncomine.org/resource/login.html) and the Gene Expression Omnibus (GEO) database (https://www.ncbi.nlm.nih.gov/geo/).

Human HNSCC cell lines (HN30, HN4, Cal27, and HN6) obtained from Shanghai Key Laboratory of Stomatology and Shanghai Research Institute of Stomatology were cultured in Dulbecco’s modified Eagle medium (DMEM) (Gibco, Carlsbad, CA, USA), and HNSCC cell lines (SCC25 and SCC9) were cultured in DMEM/F12 (Gibco, Carlsbad, CA, USA) supplemented with 10% heat-inactivated fetal bovine serum (FBS; Gibco, Carlsbad, CA, USA) at 37 °C in a humidified 5% CO_2_ atmosphere. TRIzol Reagent (Invitrogen, Carlsbad, CA, USA) was used to extract total RNA from freshly harvested cells. A PrimeScript RT-PCR Kit (TakaraBio, Otsu, Japan) was used for the reverse transcription of RNA. An ABI 7500 real-time PCR system (Applied Biosystems, Irvine, CA, USA) and SYBR Select MasterMix (Applied Biosystems, Irvine, CA, USA) were used for qRT-PCR. The primer sequences were as follows: forward 5′-CCTAAAGCCACGCTTCTTTG-3′ and reverse 5′-TGCAGGCTGGAGATCCTACT-3′ for HOXA distal transcript antisense RNA (HOTTIP); forward 5′-AGAAAGACTGAGCGTGGGA-3′ and reverse 5′-GTCATTTTGGAGGCTGGAA-3′ for LINC00460; forward 5′-CGGGCTTGTGGAAT GGTAAGC-3′ and reverse 5′-GCTTCGGCAGCACATATACTAAAAT-3′ for hsa-miR-206; forward 5′-AGGTCGGTGTGAACGGATTTG-3′ and ′and reverse 5′-TGTAGACCATGTAGTTGAGGTCA-3′ for GAPDH. The relative expression levels were estimated using the 2^−ΔΔCT^ equation.

### Statistical analysis

The expression of each RNA correlating with clinical characteristics was estimated using the Mann–Whitney rank sum test. Survival curves were plotted using the Kaplan–Meier method. Prognostic RNAs were screened using univariate and LASSO regression analyses. R software (version 3.6.0), Cytoscape software (version 3.5.1), and GraphPad Prism 7 were used to plot figures. Statistical significance was set at *p* < 0.05.

## Results

### Identification of DElncRNAs, DEmRNAs and DEmiRNAs

**Figure 2:**
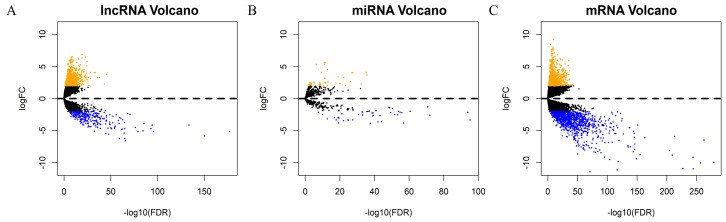
Screening of prognostic factors associated with OS. Volcano maps of (A) DElncRNA, (B) DEmiRNA, and (C) DEmRNA. The orange points represent up-regulated RNAs, blue represent down-regulated RNAs, and black represent no significant difference. DElncRNA, differentially expressed long noncoding RNA; DEmRNA, differentially expressed messenger RNA; DEmiRNA, differentially expressed microRNA; HR, hazard ratio; OS, overall survival.

Each sample from patients with HNSCC was analyzed to obtain the expression profiles of lncRNA, mRNA, and miRNA for comprehensive integrated analysis. A total of 1052 DElncRNAs, 82 DEmiRNAs, and 2001 DEmRNAs were differentially expressed between HNSCC and normal tissue samples. Of these, 766 lncRNAs (72.81%) were upregulated and 286 (27.19%) were downregulated; 873 mRNAs (43.63%) were upregulated and 1128 (56.37%) downregulated; and 44 miRNAs (53.66%) were upregulated and 38 (46.34%) were downregulated. The “gplots” package was used to plot the volcanos and heat maps of differentially expressed RNAs (Figs. 2A–2C, Figs. S1A–S1C).

### Screening of prognostic factors associated with OS

We assessed 474 HNSCC samples selected from TCGA against the following criteria: (1) has clinical characteristics data, (2) has whole RNA-seq data, and (3) OS of more than 100 days. DElncRNAs, DEmiRNAs, and DEmRNAs related to OS were screened using univariate Cox regression analysis. In total, 207 lncRNAs, 18 miRNAs, and 362 mRNAs identified by univariate Cox regression analysis (*p* < 0.05). Forthermore, 19 lncRNAs, 18 miRNAs, and 25 mRNAs were selected for the LASSO regression analysis to establish the risk signatures (DElncRNAs and DEmRNAs with *p* < 0.001, and DEmiRNAs with *p* < 0.05) (Table S1).

### Functional analysis

**Figure 3:**
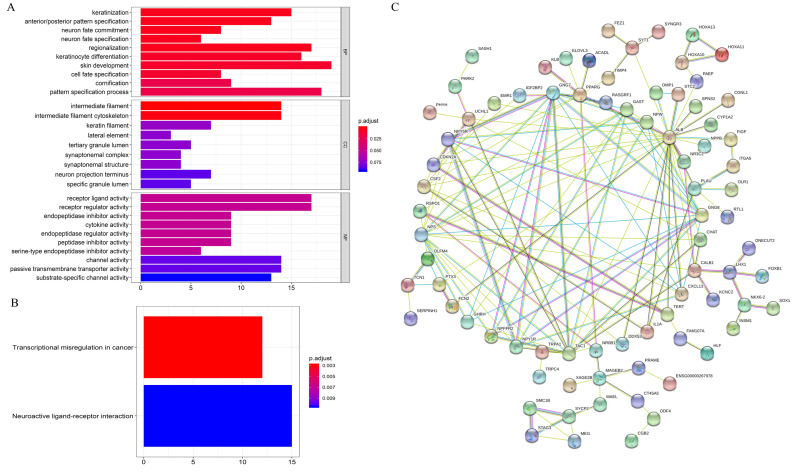
Functional annotation of overall survival-associated mRNAs. (A) GO analysis of the mRNAs, (B) KEGG pathway enriched by the hub mRNAs, (C) PPI network construction by STRING. BP, biological processes; CC, cellular component; MF, molecular function; PPI, protein–protein interaction.

GO enrichment analysis showed that on the BP, the differential expression genes were associated with “keratinization”, “anterior/posterior pattern specification”, etc. For MF, the genes were related to “receptor ligand activity”, “receptor regulator activity”, etc. CC were closely correlated with “intermediate filament”, “intermediate filament cytoskeleton”, etc (Fig. 3A). KEGG enrichment analysis revealed that mRNAs were dramatically enriched in pathways related to “transcriptional misregulation in cancer” and “neuroactive ligand–receptor interaction”, which are significantly correlated with carcinogenesis and metastasis (Fig. 3B). Further, correlations of the top 185 most significant mRNAs (*p* < 0.05) were analyzed by constructing PPI networks (Fig. 3C). The results showed that these mRNAs play key roles during the initiation and progression of HNSCC.

### Predictive model for OS

**Figure 4:**
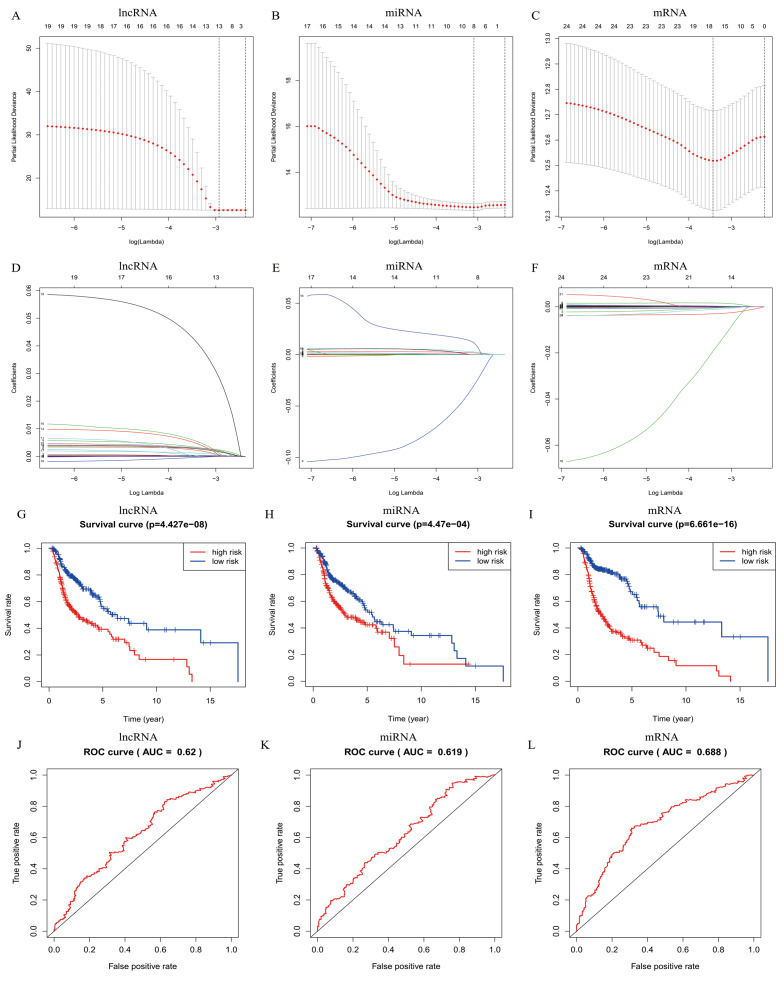
Risk signature with prognostic-associated model RNAs. (A) The process of building the signature containing (A–D) lncRNA, (B–E) miRNA, and (C–F) mRNA using LASSO are shown. Kaplan-Meier overall survival curves of the (G) lncRNA model, (H) miRNA model, and (I) mRNA model for patients in high-risk and low-risk group divided according to the risk score. ROC analysis and AUC value of the ROC curve suggesting the sensitivity and specificity for (J) lncRNA, (K) miRNA, and (L) mRNA risk signature. Log-rank *p* < 0.05 was considered statistical significance.

In total, 19 lncRNAs, 18 miRNAs, and 25 mRNAs identified in univariate Cox regression analysis were selected for the LASSO regression analysis. Of these, 13 lncRNAs (LINC00460, AL136987.1, MYOSLID, MIR9-3HG, AC073130.1, AC079160.1, LINC01305, AP002478.1, LINC02434, HOTTIP, ATP6V1B1-AS1, AC023310.4, and AL158209.1), eight miRNAs (hsa-miR-411, hsa-miR-4510, hsa-miR-410, hsa-miR-99a, hsa-miR-499a, hsa-miR-4652, hsa-miR-206 and hsa-miR-520e), and 17 mRNAs (CELSR3, STC2, ADGRD2, ZNF541, GRB14, NOSTRIN, TIMP4, HOXB9, ADPRHL1, SPINK1, PTX3, FRZB, ODF4, PLAU, OLR1, GNG7, and DTHD1) were independent prognostic factors related to OS in patients with HNSCC. Associations between each of the above-mentioned RNAs and clinicopathological features and molecular characteristics were assessed (Tables S2–S4). Based on the powerful prognostic RNAs, three risk signatures were constructed (Figs. 4A–4F). LASSO regression analysis of the 13 lncRNAs, eight miRNAs, and 17 mRNAs was conducted, and the RS was calculated according to the coefficients shown in Table 1. To evaluate the prognostic performance of the risk signature, patients with HNSCC were divided into low- and high-risk groups based on the median RS. The results suggested that patients in the high-risk group had worse prognoses than those in the low-risk group (Figs. 4G–4I). In addition, specificity and sensitivity were assessed using a time-dependent ROC curve, and the AUC values were 0.62, 0.619, and 0.688, respectively, suggesting better prediction performance (Figs. 4J–4L).

**Table 1:** The coefficients estimated by LASSO regression analysis.

DElncRNA	Coef		DEmiRNA	Coef		DEmRNA	Coef
LINC00460	2.1735E−04		has-miR-411	2.0123E−04		CELSR3	−8.2570E−05
AL136987.1	2.3890E−03		has-miR-4510	−3.0047E−02		STC2	3.8044E−05
MYOSLID	1.6926E−04		has-miR-410	1.9585E−04		ADGRD2	−6.0742E−04
MIR9-3HG	−2.8041E−05		has-miR-99a	−6.1385E−05		ZNF541	−1.2459E−04
AC073130.1	4.1471E−04		has-miR-499a	1.7026E−03		GRB14	2.7241E−04
AC079160.1	1.9663E−03		has-miR-4652	2.6325E−03		NOSTRIN	−3.0643E−03
LINC01305	−7.1734E−07		has-miR-206	1.8113E−06		TIMP4	1.6223E−03
AP002478.1	2.1455E−03		has-miR-520e	1.1725E−02		HOXB9	9.9531E−05
LINC02434	1.6812E−03					ADPRHL1	3.1718E−04
HOTTIP	4.2863E−04					SPINK1	3.3189E−04
ATP6V1B1-AS1	1.1889E−03					PTX3	1.1693E−04
AC023310.4	6.1627E−04					FRZB	−3.4147E−05
AL158209.1	2.8547E−02					ODF4	−1.9280E−02
						PLAU	5.3363E−06
						OLR1	1.1427E−05
						GNG7	−3.3920E−04
						DTHD1	−1.2034E−03

**Notes.**

DElncRNA differentially expressed long noncoding RNA DEmRNA differentially expressed messenger RNA DEmiRNA differentially expressed microRNA Coef coefficient

**Figure 5:**
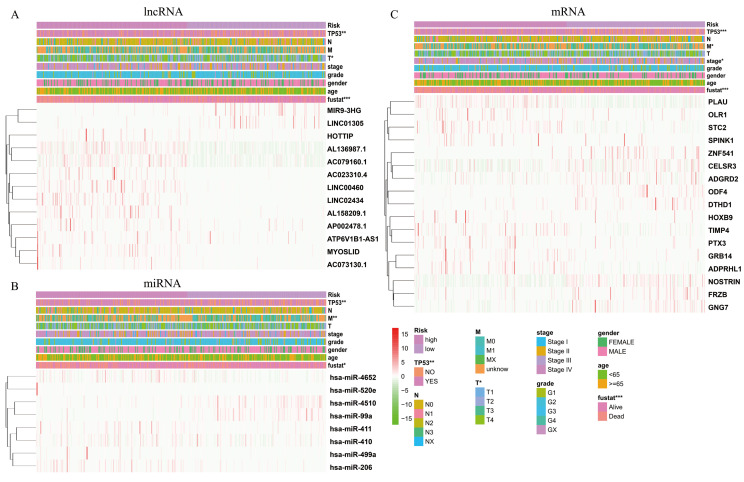
The heatmaps show the expression of three prognostic-associated model in high-risk and low-risk HNSCC. The distribution of clinicopathological features was compared between the high-risk and low-risk groups of (A) lncRNA, (B) miRNA, and (C) mRNA. * *p* < 0.05, ** *p* < 0.01 and *** *p* < 0.001.

**Figure 6:**
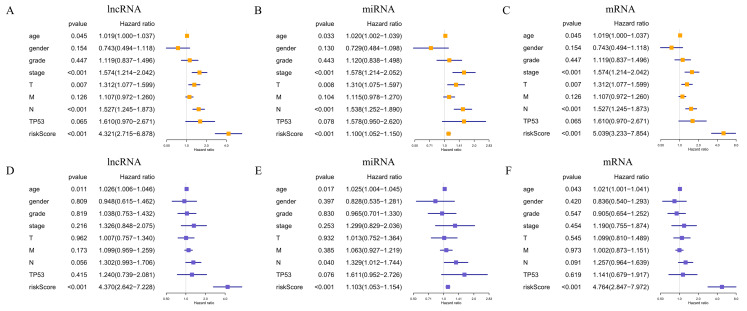
Prognostic value of the risk signature in HNSCC patients. Univariate Cox regression analysis of the associated between clinicopathological features (including riskScore) and overall survival of HNSCC patients of (A) lncRNA, (B) miRNA, and (C) mRNA. Multivariate Cox regression analysis of the associated between clinicopathological features (including riskScore) and overall survival of (D) lncRNA, (E) miRNA, and (F) mRNA. HR <1 represents RNA was negatively associated with OS, while HR >1 represents RNA was positively associated with OS. HR, hazard ratio; OS, overall survival.

The expression levels of differentially expressed RNAs related to clinicopathological characteristics, including the three risk signatures, are shown in the heat map (Figs. 5A–5C). Classification into the high-risk group was closely related to patient died status, TP53 mutation status, a high T stage, HPV negative, PNI, and disease recurrence/progression in the lncRNA-based model; to patient died status, TP53 mutation status, high M stage, and HPV negative in the miRNA-based model; and to patient died status, TP53 mutation status, high clinical stage, a high M stage, HPV negative, PNI, ECS, and disease recurrence/progression in the mRNA-based model (Figs. 5A–5C, Table 2). Thus, age, stage, T stage, N stage and the risk score were strongly related to OS in univariate analysis (Figs. 6A–6C); risk score and age, in the lncRNA and mRNA models; and risk score, age, and N stage, in the miRNA model (multivariate analysis) (Figs. 6D–6F) (*p* < 0.05). These results showed that the risk score could be regarded as an independent prognostic factor in HNSCC.

### Construction of the survival-related ceRNA network

**Figure 7:**
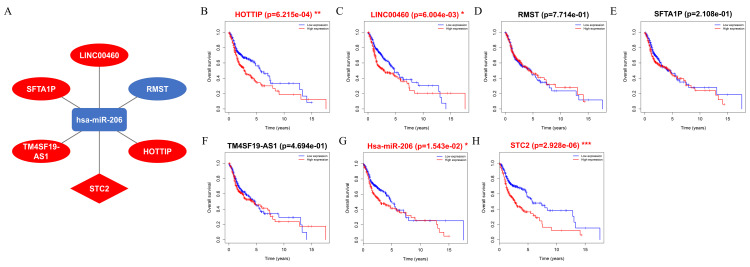
Construct ceRNA regulatory network, and the prognostic value of RNAs included in ceRNA regulatory network. (A) CeRNA network in HNSCC. Ellipse nodes represent lncRNAs; Diamond nodes represent mRNAs; rectangle nodes represent miRNAs. The red nodes represent increased level of expression, while the blue nodes represent decreased level of expression. Gray edges indicate lncRNA-miRNA-mRNA interactions. (B–H) The overall survival curves of the ceRNA-associated RNAs estimated by the Kaplan Meier plotter. * *p* < 0.05, ** *p* < 0.01 and *** *p* < 0.001.

A ceRNA network comprising DElncRNAs, DEmiRNAs, and DEmRNAs linked to HNSCC was established and visualized using Cytoscape. The results showed that five lncRNAs (HOTTIP, LINC00460, RMST, SFTA1P, and TM4SF19-AS1), one miRNA (hsa-miR-206), and one mRNA (STC2) were eligible for the ceRNA regulatory network (Fig. 7A). Moreover, the results showed that patients with HNSCC and high HOTTIP, LINC00460, miRNA-206, and STC2 expression had a poor prognosis (Figs. 7B, 7C, 7G and 7H).

### Verification of the prognostic values of ceRNA

**Figure 8:**
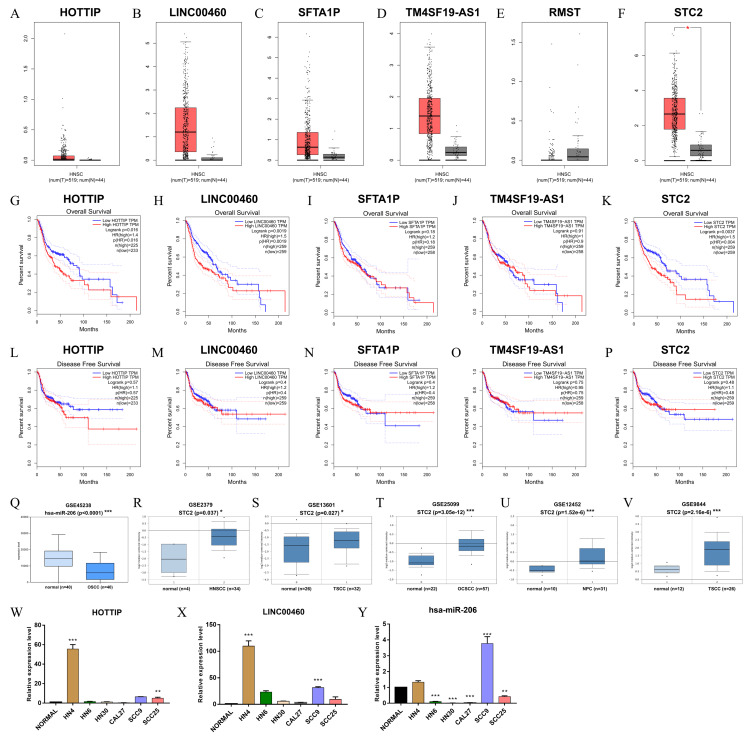
Validation of RNAs level in human HNSC. (A–F) The tangerine and gray boxes represent cancer and normal tissues, respectively. (G–K) The overall survival of lncRNA, and mRNA. (L–P) The disease-free survival of lncRNA, and mRNA. (Q) The expression of hsa-miR-206 in GEO microarray. (R–V) The expression of STC2 in five GEO microarrays. (W–Y) The transcriptional expression of HOTTIP, LINC00460 and hsa-miR-206by qRT-PCR in normal and six HNSCC cell lines. HNSC, Head and neck squamous cell carcinoma; num, Number; T, Tumor; N, Normal. * *p* < 0.05, ** *p* < 0.01 and *** *p* < 0.001.

The verified results of GEPIA showed that HOTTIP, LINC00460, and STC2 were significantly associated with OS, strengthening the reliability of our findings (Figs. 8A–8P). Notably, the expression of hsa-miR-206 (GSE45238) and STC2 (GSE2379, GSE13601, GSE25099, GSE12452 and GSE9844) in six microarrays was significantly differential expression in HNSCC tissues than in normal tissues (Figs. 8Q–8V). Furthermore, qRT-PCR results showed that HOTTIP and LINC00460 were upregulated (Figs. 8W and 8X), hsa-miR-206 was downregulated (Fig. 8Y) to varying degrees in many HNSCC cell lines (HN4, HN6, Cal27, HN30, SCC9, and SCC25).

**Table 2:** Clinicopathological features are different between high-risk and low-risk in DElncRNA, DEmiRNA and DEmRNA.

Clinicopathological features		DElncRNA			DEmiRNA			DEmRNA		
		High-risk	Low-risk	*p*	High-risk	Low-risk	*p*	High-risk	Low-risk	*p*
HPV p16	Negative	29	37	**9.11e**^−3^^**^	42	22	**1.67e**^−5^^***^	35	30	**4.60e**^−5^^***^
	Positive	4	25		4	24		2	27	
HPV ish	Negative	23	30	**8.19e**^−3^^**^	34	18	**6.87e**^−5^^***^	27	25	**2.95e**^−4^^***^
	Positive	1	17		1	16		0	18	
Perineural Invasion	Yes	93	62	0.01^*^	88	64	0.17	103	51	**8.34e**^−6^^***^
	No	80	95		87	88		73	102	
Angiolymphatic Invasion	Yes	58	51	0.73	62	44	0.25	65	44	0.12
	No	106	104		107	103		104	105	
Extracapsular spread pathologic	Gross Extension	20	12	0.34	15	16	0.22	19	13	**6.12e**^−3^^**^
	Microscopic Extension	35	35		43	26		47	22	
	No Extranodal Extension	110	116		114	110		106	120	
Lymph nodes counts	<18	43	34	0.63	41	37	1	46	31	0.20
	≥18	161	149		160	145		157	152	
Disease Free Status	Disease Free	88	138	**1.25e**^−3^^**^	105	121	0.64	73	155	**1.20e**^−8^^***^
	Recurred/Progressed	78	59		67	68		86	50	

**Notes.**

DElncRNA differentially expressed long noncoding RNA DEmRNA differentially expressed messenger RNA DEmiRNA differentially expressed microRNA

* *p* <  0.05.

** *p* <  0.01.

*** *p* <  0.001.

## Discussion

Determining the potential molecular mechanisms of the onset and development of HNSCC and new prognostic targets will help improve the survival rate of patients. To date, few studies have reported single or multiple biomarkers for HNSCC due to its multiple genetic alternations and mutations and their complicated interactions. Therefore, identification of specific and sensitive lncRNA biomarkers and prognostic targets for HNSCC is urgently needed (Guglas et al., 2017). ceRNA regulatory networks are composed of lncRNAs, miRNAs, and mRNAs and have attracted great interest in recent years with respect to their contribution to the molecular mechanisms underlying tumor development and subsequent malignant progression (Deng et al., 2015). The present study explored how the HNSCC-specific lncRNAs function as ceRNAs to regulate their target genes and constructed RNA-based prognostic models for HNSCC.

Many studies have implicated the ceRNA regulatory network comprising lncRNAs, miRNAs, and mRNAs in the development of HNSCC (Xu et al., 2019; Yin et al., 2019; Pan et al., 2019; Fang et al., 2018; Zhao et al., 2018). All the above studies are based on data from the TCGA database for the construction of a ceRNA regulatory network and to obtain survival-related RNAs of HNSCC. The present study screened HOTTIP, LINC00460, hsa-miR-206, and STC2 and, similar to the results of previous studies, a comparison with which indicated that our research methods were credible. However, these studies only assessed the relationship between signal lncRNA/miRNA/mRNA and survival and did not construct a prognostic model. Conventional methods are often directly used to screen for differentially expressed RNAs while constructing a ceRNA regulatory network. In contrast to other studies, in this study, we first analyzed differentially expressed RNAs to identify prognosis-related RNAs using univariate Cox regression analysis, and then selected the prognosis-related DElncRNAs, DEmiRNAs, and DEmRNAs for the construction of the ceRNA network. This approach ensured that all RNAs incorporated into the network were associated with survival.

Another study by Yin et al. (2019) showed that 71 lncRNAs, eight miRNAs, and 16 mRNAs were established an HNSCC-specific ceRNA network. HOTTIP was proven to be the most powerful prognostic factor and was markedly associated with clinical stage and histological grade in three patients with HNSCC. Moreover, our study used univariate Cox regression analysis and LASSO analysis to establish RNA-based risk signatures. Compared with multivariate Cox regression analysis, LASSO analysis may be more beneficial in reducing the occurrence of data model overfitting. However, a previous study identified only lncRNAs and mRNA prognostic signatures among the ceRNA network and could not establish a miRNA-based model. Forthermore, we analyzed the relationship between the prognostic model and different clinicopathological features, and thereby strictly explained the predictive role of risk signatures. More importantly, our study not only established three prognostic models consisting of prognostic RNAs, but also assessed the relationship between these prognostic models and conventional clinicopathological features (including TNM stage and histological grade) and specific HNSCC clinicopathological features (including TP53 mutation status, ALI, HPV status, PNI, and ECS).

Previous publications have focused in part on ceRNAs in laryngeal squamous cell carcinoma (LSCC) (Kong et al., 2019; Liu & Ye, 2019; Gao et al., 2019; Zhao et al., 2019; Sun et al., 2018; Feng et al., 2016), tongue squamous cell carcinoma (TSCC) (Sturgis & Cinciripini, 2007), and nasopharyngeal carcinoma (NPC) (Fullerton et al., 2020; Agrawal et al., 2011). These studies have identified differentially expressed RNAs by lncRNA and mRNA integrated microarrays of cancer tissue or by immunohistochemistry (Gao et al., 2019; Zhao et al., 2019; Sun et al., 2018; Feng et al., 2016). The different key RNAs screened may be attributed to the patients included from different databases and the different tumor types. The evaluation ability was 0.62 for the lncRNA-based model, 0.619 for the miRNA-based model, and 0.688 for the mRNA-based model.

Importantly, while establishing prognosis-related prediction models, we also analyzed the relationship between the three prognostic models and clinicopathological features, including the relationship between the expression of each independent RNA in the model and clinicopathological features. This has, to the best of our knowledge, never been explored. Our results showed that the three prognostic models significantly correlated with clinicopathological features, such as OS, DFS, TP53 mutation status, and HPV status. Furthermore, the RS of the three risk signatures as an independent predictor of prognosis plays an important role in prognostic prediction in HNSCC. Notably, HPV is regarded as a crucial risk factor for the development of oropharyngeal cancer (Sturgis & Cinciripini, 2007). New studies have shown that patients with HPV-positive and HPV-negative oropharyngeal cancer have dramatically different rates of both head and neck carcinoma mortality and competing-cause mortality. Compared with HPV-positive patients, HPV-negative patients have a higher risk of two-year cumulative incidence of all-cause mortality and a lower risk of both head and neck carcinoma-specific mortality and competing-cause mortality (*p* < 0.0001) (Fullerton et al., 2020). Therefore, we believe that the HPV status is a critical determinant in oropharyngeal cancer. TP53 mutation was observed in 60% to 80% of HPV-negative HNSCC patients and was associated with poor prognosis, increased resistance to standard therapy (mainly cisplatin and radiation), and the shortest time to distant metastases (Agrawal et al., 2011; Stransky et al., 2011; Neskey et al., 2015). The significance of TP53 mutations is in predicting the efficacy of chemotherapy, targeted therapy, and radiotherapy.

In our KEGG pathway analysis, the prognostic mRNAs were significantly enriched in “Transcriptional misregulation in cancer” and “Neuroactive ligand–receptor interaction”. The pathways related to “Transcriptional misregulation in cancer” included the P53 signaling pathway, renal cell carcinoma, thyroid cancer, and acute myeloid leukemia. Xu et al. (2019) reported the same KEGG pathways to be enriched in HNSCC.

We further validated the RNAs in the ceRNA regulatory network using the TCGA database, both in vivo and in vitro. HOTTIP and LINC00460 were significantly associated with OS validated by GTEx data and several gene microarrays. They also showed varying degrees of upregulation in six HNSCC cell lines. HOTTIP is a newly identified lncRNA encoded by chromosome 7p15.2 located at the 5 ′-end of the HOXA cluster (Wang et al., 2011). Many studies have reported that higher expression of HOTTIP is associated with positive lymph node metastasis and unfavorable prognosis in various cancer types (Lian et al., 2016; Chen et al., 2017). Our previous studies have confirmed that HOTTIP is also involved in the construction of a ceRNA regulatory network in renal clear-cell carcinoma (Wang et al., 2018). Zhang et al. (2015) reported that the overexpression of HOTTIP is an independent poor prognostic factor for patients with TSCC. Xue et al. (2019) showed that the downregulation of LINC00460 can facilitate cancer cell apoptosis and autophagy in HNSCC. Our previous studies have confirmed that the lncRNA signature and tumor grade are independent prognostic factors and that the 3-lncRNA (KTN1-AS1, LINC00460, and RP5-894A10.6) signature could be a novel prognostic biomarker for HNSCC (Cao et al., 2017). Jiang et al. (2019) proposed that LINC00460 could enhance the proliferation and metastasis of HNSCC cells and promote EMT by accelerating PRDX1 entry into the nucleus. LINC00460 contributes to the progression of NPC by sponging miR-149-5p to upregulate IL6 (Kong et al., 2018).

In this study, only one prognostic miRNA (hsa-miR-206) involved in ceRNAs network. In terms of hsa-miR-206, it has been reported to be downregulated and associated with tumor progression and worse prognosis in several malignancies such as gastric cancer, osteosarcoma and colorectal carcinoma (Arigami et al., 2013; Bao et al., 2013; Liu et al., 2017). In HNSCC, it was demonstrated that hsa-miR-206 plays a critical role progression by targeting HDAC6 via PTEN/AKT/mTOR pathway, which might be a potential target for diagnostic and therapeutic (Vickers et al., 2012). The target prognostic mRNA of hsa-miR-206 was STC2 in ceRNAs regulatory network in present study. STC2 (Stanniocalcin 2) is a secreted glycoprotein with important functions in gastric cancer, which could be a powerful marker of poor prognosis (Yang et al., 2013; Yang et al., 2017). Yang et al. concluded that STC2 may promote HNSCC metastasis via PI3K/AKT/Snail signaling axis. Targeted therapy against STC2 may become a novel strategy to effectively treat patients with metastatic HNSCC (Yokobori et al., 2010).

This study has various advantages and limitations. The advantage of bioinformatics technology is that it can provide novel insights into the potential molecular mechanisms involved in the development and progression of HNSCC (Yu et al., 2014). However, a well-designed and verified classifier model is needed to ensure the reliability of our findings. The highlights of this study are as follows: First, we established three multi-RNA-based prognostic models and a prognostic ceRNA regulatory network for HNSCC, all of which can increase the effectiveness of prognosis prediction. Second, we comprehensively analyzed the relationship between the prognosis model and each RNA included in the prediction model and conventional clinicopathological features (including TNM stage and histological grade) and specific HNSCC clinicopathological features (including TP53 mutation status, ALI, HPV status, PNI, and ECS) to further verify the significant correlation with survival and clinical therapeutic outcomes. lncRNAs can regulate gene expression at several levels and are involved in the evolution and progression of many cancers. However, the present study only identified one regulatory mechanism. Therefore, further clinical investigations and scientific research are required to validate these conclusions.

## Conclusions

In conclusion, the present study analyzed an independent cohort of patients with HNSCC from the TCGA database, constructed a ceRNA regulatory network, and established three multi-RNA-based prognostic models with potential prognostic value, and the findings may provide a basis for further experimental and clinical research.

##  Supplemental Information

10.7717/peerj.9701/supp-1 Supplemental Information 1 Univariate Cox regression analysis of RNAs involved in risk signature

10.7717/peerj.9701/supp-2 Supplemental Information 2 Relationships between the expression of lncRNAs and clinicopathological features and molecular characteristics in HNSCC patients

10.7717/peerj.9701/supp-3 Supplemental Information 3 Relationships between the expression of miRNAs and clinicopathological features and molecular characteristics in HNSCC patients

10.7717/peerj.9701/supp-4 Supplemental Information 4 Relationships between the expression of mRNAs and clinicopathological features and molecular characteristics in HNSCC patients

10.7717/peerj.9701/supp-5 Supplemental Information 5 Heatmaps of (A) DElncRNA, (B) DEmiRNA, and (C) DEmRNA The red points represent up-regulated RNAs, green represent down-regulated RNAs, and black represent no significant difference.

10.7717/peerj.9701/supp-6 Supplemental Information 6 qRT-PCR Raw data of miR-206

## Additional Information and Declarations

### Data Availability

The following information was supplied regarding data availability: Data is available at TCGA, search terms: TCGA, Project: TCGA–HNSC, Diease Type: squamous cell neoplasms.
https://portal.gdc.cancer.gov/repository?facetTab=casesfilters=op%3A"and"%2C"content"%3A[op%3A"in"%2C"content"%3Afield%3A"cases.project.project_id"%2C"value"%3A[TCGA-HNSC]]
Data is available at NCBI GEO: GSE45238, GSE2379, GSE13601, GSE25099, GSE9844, GSE12452.
