# Supplementary material for: A prognostic long non-coding RNA-associated competing endogenous RNA network in head and neck squamous cell carcinoma
Source: PeerJ. 2020 Sep 15;8:e9701. doi: 10.7717/peerj.9701 (PMC7500352; doi:10.7717/peerj.9701)
Supplement: Supplemental Information 1 [file peerj-08-9701-s001.docx]

| Supplement Table 1 Univariate Cox regression analysis of RNAs involved in risk signatures | | | | | | | | | | | | | |
| --- | --- | --- | --- | --- | --- | --- | --- | --- | --- | --- | --- | --- | --- |
| **DElncRNA** | | | |  | **DEmiRNA** | | | |  | **DEmRNA** | | | |
|  | HR | z | p-value |  |  | HR | z | p-value |  |  | HR | z | p-value |
| AC083967.1 | 1.1930 | 4.6156 | 3.9205E-06 |  | hsa-mir-410 | 1.2067 | 3.7177 | 2.0108E-04 |  | CELSR3 | 0.7996 | -4.7505 | 2.0300E-06 |
| AL158209.1 | 1.3242 | 4.3464 | 1.3839E-05 |  | hsa-mir-411 | 1.2015 | 3.5841 | 3.3822E-04 |  | STC2 | 1.2362 | 4.2129 | 2.5200E-05 |
| LINC02434 | 1.1168 | 3.9373 | 8.2406E-05 |  | hsa-mir-381 | 1.2049 | 3.5737 | 3.5194E-04 |  | ADGRD2 | 0.8449 | -4.0713 | 4.6800E-05 |
| AL136987.1 | 1.1714 | 3.7910 | 1.5002E-04 |  | hsa-mir-99a | 0.8489 | -3.4580 | 5.4420E-04 |  | ZNF541 | 0.8871 | -4.0558 | 5.0000E-05 |
| AC007879.3 | 1.2520 | 3.7559 | 1.7274E-04 |  | hsa-mir-499a | 1.1377 | 3.1538 | 1.6117E-03 |  | RSPO1 | 0.8575 | -4.0077 | 6.1300E-05 |
| ATP6V1B1-AS1 | 1.2079 | 3.7221 | 1.9759E-04 |  | hsa-let-7c | 0.8568 | -2.9384 | 3.2996E-03 |  | GRB14 | 1.1494 | 3.9368 | 8.2600E-05 |
| LINC00460 | 1.1199 | 3.7163 | 2.0219E-04 |  | hsa-mir-521-1 | 1.2292 | 2.7101 | 6.7271E-03 |  | SYT14 | 1.1230 | 3.9158 | 9.0100E-05 |
| HOTTIP | 1.1828 | 3.6958 | 2.1918E-04 |  | hsa-mir-1911 | 1.0903 | 2.6548 | 7.9351E-03 |  | NOSTRIN | 0.6789 | -3.8786 | 1.0505E-04 |
| LINC02450 | 1.1551 | 3.6489 | 2.6339E-04 |  | hsa-mir-548f-1 | 1.1141 | 2.5261 | 1.1534E-02 |  | TIMP4 | 1.1744 | 3.8459 | 1.2012E-04 |
| MYOSLID | 1.1687 | 3.6099 | 3.0637E-04 |  | hsa-mir-299 | 1.1266 | 2.2915 | 2.1936E-02 |  | HOXB9 | 1.1062 | 3.7286 | 1.9253E-04 |
| MIR9-3HG | 0.8842 | -3.5790 | 3.4489E-04 |  | hsa-mir-206 | 1.0372 | 2.2405 | 2.5059E-02 |  | ADPRHL1 | 1.2045 | 3.7125 | 2.0523E-04 |
| AC023310.4 | 1.1252 | 3.5783 | 3.4583E-04 |  | hsa-mir-4510 | 0.8017 | -2.2313 | 2.5659E-02 |  | SPINK1 | 1.1245 | 3.6506 | 2.6167E-04 |
| AC073130.1 | 1.1871 | 3.5599 | 3.7100E-04 |  | hsa-mir-520e | 1.2292 | 2.2151 | 2.6755E-02 |  | PTX3 | 1.1222 | 3.6428 | 2.6964E-04 |
| LINC01305 | 0.8949 | -3.5232 | 4.2633E-04 |  | hsa-mir-1-1 | 1.0440 | 2.1792 | 2.9314E-02 |  | ZFR2 | 0.8983 | -3.5057 | 4.5533E-04 |
| AP002478.1 | 1.1591 | 3.5232 | 4.2636E-04 |  | hsa-mir-133b | 1.0413 | 2.0840 | 3.7160E-02 |  | FRZB | 0.8547 | -3.4973 | 4.6997E-04 |
| AL355596.1 | 1.1687 | 3.4233 | 6.1858E-04 |  | hsa-mir-4652 | 1.0892 | 2.0436 | 4.0992E-02 |  | ODF4 | 0.7873 | -3.4915 | 4.8023E-04 |
| LINC01234 | 1.0714 | 3.3294 | 8.7039E-04 |  | hsa-mir-520c | 1.1599 | 2.0234 | 4.3028E-02 |  | SPIB | 0.8979 | -3.4550 | 5.5030E-04 |
| AC079160.1 | 1.1186 | 3.3189 | 9.0372E-04 |  | hsa-mir-1-2 | 1.0402 | 1.9853 | 4.7114E-02 |  | PLAU | 1.2224 | 3.4512 | 5.5810E-04 |
| LINC02015 | 1.1229 | 3.3170 | 9.0993E-04 |  |  |  |  |  |  | ITGA5 | 1.2144 | 3.4389 | 5.8409E-04 |
|  |  |  |  |  |  |  |  |  |  | OLR1 | 1.1337 | 3.4099 | 6.4988E-04 |
|  |  |  |  |  |  |  |  |  |  | CFAP73 | 0.8188 | -3.4035 | 6.6526E-04 |
|  |  |  |  |  |  |  |  |  |  | TRIML2 | 1.0807 | 3.3803 | 7.2401E-04 |
|  |  |  |  |  |  |  |  |  |  | GNG7 | 0.8335 | -3.3641 | 7.6806E-04 |
|  |  |  |  |  |  |  |  |  |  | DTHD1 | 0.8770 | -3.3593 | 7.8131E-04 |
|  |  |  |  |  |  |  |  |  |  | CLEC3B | 0.8324 | -3.3097 | 9.3411E-04 |

DElncRNA, differentially expressed long noncoding RNA; DEmRNA, differentially expressed messenger RNA; DEmiRNA, differentially expressed microRNA; HR, hazard ratio; OS, overall survival. HR> 1, lncRNA was negatively associated with OS. HR< 1, lncRNA was positively associated with OS.
