# Supplementary material for: A prognostic long non-coding RNA-associated competing endogenous RNA network in head and neck squamous cell carcinoma
Source: PeerJ. 2020 Sep 15;8:e9701. doi: 10.7717/peerj.9701 (PMC7500352; doi:10.7717/peerj.9701)
Supplement: Supplemental Information 2 [file peerj-08-9701-s002.docx]

| Clinicopathological features | | **LINC00460** | | **AL136987.1** | | **MYOSLID** | | **MIR9.3HG** | | **AC073130.1** | |
| --- | --- | --- | --- | --- | --- | --- | --- | --- | --- | --- | --- |
|  |  | median (95% CI) | p | median (95% CI) | p | median (95% CI) | p | median (95% CI) | p | median (95% CI) | p |
| Age | <65 | 34.26 (77.71,113.46) | 0.191 | 13.16 (14.66, 18.31) | 0.171 | 128.61 (194.26,287.03) | 0.342 | 137.12 (427.13,652.10) | 0.027 | 3.27 (4.91,9.36) | 0.858 |
|  | ≥65 | 41.12 (65.75,98.31) |  | 15.42 (15.93,21.49) |  | 105.92 (143.40,218.91) |  | 99.12 (214.64,418.03) |  | 3.40 (2.17,12.39) |  |
| Gender | F | 50.89 (69.73,110.81) | 0.071 | 15.45 (14.69, 19.83) | 0.441 | 124.36 (148.98,227.12) | 0.333 | 88.87 (152.08,357.47) | 0.002 | 3.98 (4.53,7.30) | 0.104 |
|  | M | 33.51 (74.94,106.19) |  | 13.43 (15.48, 19.29) |  | 110.03 (187.60,270.55) |  | 138.22 (425.17,628.51) |  | 2.93 (4.49,10.84) |  |
| T | T1-2 | 33.44 (56.59,88.33) | 0.106 | 13.61 (13.81, 18.42) | 0.243 | 105.92 (132.17,182.07) | 0.003 | 115.32 (332.03,581.94) | 0.050 | 3.38 (4.46,7.63) | 0.463 |
|  | T3-4 | 47.37 (86.17,126.09) |  | 15.00 (16.16, 20.19) |  | 137.97 (204.94,05.83) |  | 101.17 (234.21,398.19) |  | 3.73 (4.29,9.13) |  |
| N | N0 | 30.87 (57.47,99.65) | 0.197 | 14.16 (16.17, 21.20) | 0.074 | 127.03 (166.49,242.97) | 0.662 | 110.47 (273.85,497.08) | 0.703 | 3.17 (4.37,7.19) | 0.870 |
|  | N1-3 | 51.02 (81.62,120.04) |  | 13.43 (13.78, 17.66) |  | 120.03 (179.97,286.92) |  | 106.60 (255.82,418.24) |  | 3.59 (4.16,9.83) |  |
| Stage | I-II | 34.52 (54.11,97.40) | 0.598 | 13.87 (13.81, 20.14) | 0.870 | 112.05 (134.46,199.40) | 0.295 | 105.16 (234.25,577.22) | 0.928 | 3.61 (4.06,8.49) | 0.646 |
|  | III-IV | 43.82 (82.02,115.52) |  | 14.16 (15.79, 19.35) |  | 129.02 (194.25,276.82) |  | 106.21 (275.70,429.32) |  | 3.52 (4.61,8.61) |  |
| Grade | I-II | 38.14 (78.07,108.70) | 0.168 | 13.51 14.90, 18.46) | 0.385 | 128.80 (190.75,255.89) | <0.001 | 105.37 (273.15,434.80) | 0.008 | 3.49 (5.04,6.99) | 0.323 |
|  | III-IV | 36.58 (57.02,98.19) |  | 15.69 (15.28, 21.56) |  | 76.80 (122.57,305.67) |  | 179.30 (443.60,819.79) |  | 2.99 (1.59,20.68) |  |
| TP53 | W | 19.37 (41.07,93.47) | <0.001 | 12.60 (12.52, 17.71) | 0.093 | 56.66 (79.03,216.99) | <0.001 | 275.64 (868.89,1376.89) | <0.001 | 2.03 (1.35,11.54) | <0.001 |
|  | MU | 49.90 (84.69,114.00) |  | 14.12 (16.27, 20.07) |  | 139.62 (209.98,283.18) |  | 101.94 (177.85,240.00) |  | 3.84 (4.81,10.17) |  |
| PNI | NO | 21.45 (51.92,93.68) | <0.001 | 13.69 (14.82, 19.63) | 0.955 | 95.47 (136.68,221.68) | <0.001 | 141.01 (349.93,579.52) | <0.001 | 2.38 (2.17,12.57) | <0.001 |
|  | YES | 63.43 (91.93,136.09) |  | 13.98 (14.55, 19.57) |  | 179.78 (215.53,358.39) |  | 89.94 (145.46,373.35) |  | 4.93 (4.95,12.65) |  |
| ALI | NO | 37.77 (70.18,108.69) | 0.727 | 13.05 (14.86, 19.13) | 0.839 | 131.22 (183.67,270.36) | 0.159 | 107.26 (258.64,442.13) | 0.448 | 3.38 (3.58,11.88) | 0.56 |
|  | YES | 38.80 (66.14,121.60) |  | 14.27 (13.93, 20.40) |  | 118.13 (142.43,239.59) |  | 112.75 (247.77,569.40) |  | 3.70 (4.23,.88) |  |
| ECS | NO | 33.58 (63.63,98.16) | 0.076 | 13.18 (14.91, 18.99) | 0.471 | 117.47 (162.59,232.65) | 0.252 | 106.06 (214.83,352.82) | 0.807 | 3.44 (3.92,11.93) | 0.806 |
|  | YES | 64.13 (82.31,143.29) |  | 14.10 (12.52, 18.21) |  | 155.96 (201.24,416.28) |  | 94.40 (238.77,560.74) |  | 3.76 (1.76,13.43) |  |
| HPVp16 | Neg | 28.61 (38.60,88.47) | <0.001 | 9.76 (11.94, 19.87) | 0.501 | 106.40 (145.08,332.49) | <0.001 | 144.88 (172.24,318.47) | <0.001 | 3.56 (-0.04,27.09) | 0.017 |
|  | Pos | 3.49 (2.83,55.22) |  | 8.35 (7.15, 19.33) |  | 19.56 (17.72,85.49) |  | 1593.45 (1274.33,2456.06) |  | 1.84 (1.39,3.95) |  |
| HPVish | Neg | 29.51 (35.82,86.72) | <0.001 | 7.32 (11.56, 20.94) | 0.818 | 128.94 (144.43,369.00) | <0.001 | 134.59 (149.18,314.06) | <0.001 | 3.56 (3.25,11.53) | 0.002 |
|  | Pos | 3.18 (2.71,11.82) |  | 9.70 (5.73, 23.40) |  | 18.78 (10.14,54.76) |  | 1969.08 (1601.08,3265.00) |  | 1.07 (0.74,2.70) |  |
| LN | <18 | 33.57 (54.01,102.61) | 0.499 | 13.22 (12.00, 19.00) | 0.379 | 107.32 (125.33,237.74) | 0.185 | 114.17 (246.84,594.35) | 0.147 | 3.59 (3.87,8.61) | 0.710 |
|  | ≥18 | 42.01 (80.76,114.51) |  | 13.98 (15.33, 19.17) |  | 131.64 (204.98,294.05) |  | 106.57 (247.54,387.67) |  | 3.42 (4.51,11.47) |  |
| DFS | DF | 31.65 (60.50,95.48) | 0.107 | 11.90 (13.13, 17.11) | 0.314 | 95.38 (143.51,213.88) | 0.003 | 152.40 (482.21,758.71) | 0.002 | 2.72 (3.85,6.17) | 0.133 |
|  | R/P | 39.24 (77.96,132.26) |  | 13.48 (14.25, 19.63) |  | 128.62 (191.60,321.83) |  | 100.50 (221.29,470.05) |  | 3.65 (2.49,15.74) |  |
| Status | Alive | 30.74 (62.23,92.28) | 0.001 | 12.35 (14.18, 17.79) | 0.017 | 100.82 (160.28,228.72) | 0.002 | 141.01 (442.65,664.00) | <0.001 | 2.78 (4.14,6.10) | 0.001 |
|  | Died | 60.37 (89.85,135.02) |  | 17.09 (16.77, 22.43) |  | 156.72 (193.78,323.39) |  | 101.17 (187.31,399.35) |  | 4.76 (4.63,16.37) |  |

Supplement Table 2 Relationships between the expression of lncRNAs and clinicopathological features in HNSCC patients

| Clinicopathological features | | **AC079160.1** | | **LINC01305** | | **AP002478.1** | | **LINC02434** | | **HOTTIP** | |
| --- | --- | --- | --- | --- | --- | --- | --- | --- | --- | --- | --- |
|  |  | median (95% CI) | p | median (95% CI) | p | median (95% CI) | p | median (95% CI) | p | median (95% CI) | p |
| Age | <65 | 20.42 (24.10,30.51) | 0.293 | 10.43 (44.84,91.76) | 0.023 | 1.93 (5.03,8.55) | 0.433 | 4.69 (18.24,29.70) | 0.523 | 1.41 (2.87,6.20) | 0.125 |
|  | ≥65 | 22.76 (26.28,35.62) |  | 6.77 (23.02,58.87) |  | 2.38 (4.00,6.84) |  | 6.01 (17.60,33.54) |  | 2.00 (3.32,5.27) |  |
| Gender | F | 24.35 (25.49,35.34) | 0.267 | 4.11 (9.22,33.13) | <0.001 | 2.19 (3.48,9.65) | 0.961 | 6.01 (15.85,32.59) | 0.894 | 1.34 (1.63,5.60) | 0.129 |
|  | M | 20.30 (24.88,31.23) |  | 11.03 (49.70,92.18) |  | 2.00 (4.93,7.42) |  | 4.82 (19.11,30.35) |  | 1.72 (3.42,6.07) |  |
| T | T1-2 | 14.21 (20.08,29.60) | 0.001 | 7.88 (41.42,89.77) | 0.600 | 1.60 (3.57,6.81) | 0.049 | 4.15 (11.95,22.36) | 0.180 | 1.20 (2.63,4.89) | 0.366 |
|  | T3-4 | 25.04 (28.16,35.29) |  | 7.69 (20.56,39.12) |  | 2.38 (5.31,9.18) |  | 6.11 (23.00,37.69) |  | 1.61 (3.12,6.85) |  |
| N | N0 | 21.76 (24.65,33.19) | 0.594 | 8.25 (20.66,53.04) | 0.846 | 1.63 (4.16,7.62) | 0.098 | 4.26 (15.39,30.34) | 0.368 | 1.18 (2.07,7.25) | 0.230 |
|  | N1-3 | 20.28 (25.14,33.66) |  | 7.84 (33.47,68.07) |  | 2.38 (5.02,9.40) |  | 6.23 (21.33,36.70) |  | 1.51 (2.95,5.48) |  |
| Stage | I-II | 17.73 (19.46,31.20) | 0.127 | 5.44 (14.56,57.25) | 0.150 | 1.50 (2.63,5.62) | 0.013 | 3.98 (9.97,23.34) | 0.231 | 1.11 (2.01,5.62) | 0.139 |
|  | III-IV | 22.66 (27.03,33.69) |  | 8.16 (29.29,53.99) |  | 2.29 (5.42,8.84) |  | 6.23 (21.91,34.31) |  | 1.56 (3.18,6.20) |  |
| Grade | I-II | 20.63 (25.4,31.46) | 0.724 | 6.11 (23.35,56.04) | <0.001 | 2.20 (4.73,7.64) | 0.396 | 6.60 (20.56,31.79) | 0.025 | 1.33 (2.54,3.79) | 0.016 |
|  | III-IV | 22.29 (22.32,33.72) |  | 15.89 (54.25,115.55) |  | 1.96 (4.24,9.40) |  | 3.09 (11.29,27.33) |  | 2.87 (3.79,11.77) |  |
| TP53 | W | 13.65 (18.73,29.59) | 0.009 | 17.07 (101.60,210.15) | <0.001 | 0.99 (1.70,4.16) | <0.001 | 2.03 (12.75,28.43) | 0.034 | 1.33 (1.91,4.42) | 0.094 |
|  | MU | 22.47 (27.20,33.39) |  | 6.48 (16.08,25.45) |  | 2.82 (5.92,9.11) |  | 6.13 (20.26,31.56) |  | 1.84 (3.50,6.41) |  |
| PNI | NO | 22.19 (26.22,35.39) | 0.363 | 11.97 (40.08,85.89) | 0.013 | 2.04 (5.09,9.11) | 0.903 | 2.55 (12.01,23.22) | 0.029 | 1.40 (2.70,7.43) | 0.980 |
|  | YES | 18.91 (23.17,32.97) |  | 5.36 (15.23,43.00) |  | 2.21 (3.56,8.38) |  | 6.28 (20.56,38.83) |  | 1.32 (2.83,6.57) |  |
| ALI | NO | 20.02 (23.66,31.28) | 0.613 | 5.85 (24.06,52.67) | 0.402 | 2.08 (4.04,6.86) | 0.381 | 4.22 (15.82,29.56) | 0.767 | 1.21 (2.32,6.43) | 0.031 |
|  | YES | 22.66 (24.15,35.51) |  | 8.85 (26.49,86.05) |  | 2.34 (3.83,10.44) |  | 4.72 (16.97,37.57) |  | 1.98 (3.12,7.94) |  |
| ECS | NO | 19.72 (24.09,31.80) | 0.541 | 5.99 (24.47,55.33) | 0.205 | 1.82 (4.21,7.07) | 0.012 | 4.52 (14.90,26.47) | 0.167 | 1.24 (2.51,6.21) | 0.201 |
|  | YES | 23.38 (22.84,34.51) |  | 8.38 (15.94,119.05) |  | 3.00 (5.16,13.28) |  | 6.26 (23.21,50.32) |  | 1.59 (2.74,7.91) |  |
| HPVp16 | Neg | 21.79 (22.19,35.86) | 0.076 | 5.98 (11.44,30.09) | <0.001 | 2.62 (3.56,8.17) | 0.030 | 2.95 (7.85,34.02) | 0.082 | 1.20 (1.22,8.31) | 0.090 |
|  | Pos | 4.46 (7.07,37.36) |  | 244.12 (209.31,476.86) |  | 0.96 (-0.67,6.67) |  | 0.85 (-33.7,1106.06) |  | 2.04 (1.79,7.59) |  |
| HPVish | Neg | 21.06 (18.80,31.44) | 0.096 | 6.01 (10.77,27.51) | <0.001 | 2.36 (2.43,8.75) | 0.014 | 2.50 (6.28,37.38) | 0.003 | 2.20 (1.38,10.11) | 0.615 |
|  | Pos | 1.07 (-12.70,48.92) |  | 336.37 (209.34,805.30) |  | 0.87 (0.46,1.66) |  | 0.01 (-0.24,0.97) |  | 1.48 (0.65,5.17) |  |
| LN | <18 | 21.70 (25.43,41.53) | 0.310 | 10.31 (18.14,55.07) | 0.493 | 1.87 (2.57,6.69) | 0.308 | 4.97 (13.47,44.70) | 0.536 | 1.84 (1.16,10.42) | 0.759 |
|  | ≥18 | 20.28 (24.57,30.84) |  | 6.67 (31.72,71.92) |  | 2.22 (5.41,8.81) |  | 6.26 (19.77,30.49) |  | 1.32 (2.87,5.04) |  |
| DFS | DF | 18.76 (21.17,28.05) | 0.103 | 12.68 (53.50,110.63) | 0.013 | 1.73 (3.89,6.79) | 0.232 | 4.50 (14.92,27.51) | 0.231 | 1.20 (2.06,5.58) | 0.148 |
|  | R/P | 20.48 (24.66,34.90) |  | 7.69 (16.21,71.82) |  | 2.51 (4.12,8.40) |  | 6.13 (23.65,45.36) |  | 1.67 (3.25,7.75) |  |
| Status | Alive | 20.08 (22.80,28.95) | 0.019 | 11.98 (54.32,104.16) | <0.001 | 1.83 (4.08,6.48) | 0.098 | 4.66 (17.15,28.73) | 0.747 | 1.40 (2.72,6.01) | 0.304 |
|  | Died | 24.71 (28.36,38.00) |  | 5.28 (14.07,29.73) |  | 2.59 (5.34,10.43) |  | 6.02 (19.26,34.78) |  | 1.90 (3.39,5.75) |  |

| Clinicopathological features | | **ATP6V1B1-AS1** | | **AC023310.4** | | **AL158209.1** | |
| --- | --- | --- | --- | --- | --- | --- | --- |
|  |  | median (95% CI) | p | median (95% CI) | p | median (95% CI) | p |
| Age | <65 | 1.77 (2.96,4.81) | 0.733 | 2.22 (8.13,17.13) | 0.040 | 0.93 (1.40,2.28) | 0.815 |
|  | ≥65 | 1.77 (2.92,5.24) |  | 3.62 (11.42,32.79) |  | 0.99 (1.11,1.87) |  |
| Gender | F | 1.72 (2.56,4.47) | 0.471 | 3.14 (8.90,26.58) | 0.419 | 0.75 (0.94,1.80) | 0.454 |
|  | M | 1.81 (3.20,5.04) |  | 2.46 (9.76,21.75) |  | 0.98 (1.45,2.23) |  |
| T | T1-2 | 1.58 (2.25,4.53) | 0.070 | 2.15 (4.58,25.53) | 0.033 | 0.97 (1.33,2.49) | 0.502 |
|  | T3-4 | 2.06 (3.42,5.58) |  | 2.94 (12.16,24.57) |  | 0.91 (1.25,2.08) |  |
| N | N0 | 1.69 (2.50,4.85) | 0.109 | 2.22 (6.29,14.97) | 0.194 | 0.93 (1.19,2.34) | 0.960 |
|  | N1-3 | 2.07 (3.19,5.22) |  | 2.75 (12.83,33.00) |  | 0.96 (1.38,2.35) |  |
| Stage | I-II | 1.69 (1.75,5.45) | 0.207 | 1.84 (2.93,9.58) | 0.009 | 1.08 (1.09,2.74) | 0.697 |
|  | III-IV | 2.02 (3.34,5.15) |  | 2.89 (12.91,27.17) |  | 0.93 (1.33,1.98) |  |
| Grade | I-II | 1.65 (2.78,3.95) | 0.191 | 2.64 (10.72,23.07) | 0.963 | 1.00 (1.54,2.34) | 0.005 |
|  | III-IV | 2.34 (3.37,8.19) |  | 2.81 (6.23,23.08) |  | 0.70 (0.71,1.30) |  |
| TP53 | W | 1.02 (1.49,4.67) | 0.002 | 2.13 (5.20,16.84) | 0.053 | 0.11 (0.74,1.50) | 0.016 |
|  | MU | 2.02 (3.47,5.12) |  | 2.87 (11.88,24.72) |  | 1.03 (1.51,2.30) |  |
| PNI | NO | 1.82 (2.82,5.35) | 0.370 | 2.92 (8.68,28.82) | 0.072 | 0.84 (1.15,2.09) | 0.659 |
|  | YES | 1.79 (2.67,5.01) |  | 1.91 (3.87,15.35) |  | 1.01 (1.28,2.53) |  |
| ALI | NO | 1.88 (2.94,5.43) | 0.630 | 2.57 (7.38,24.50) | 0.538 | 0.91 (1.46,2.63) | 0.834 |
|  | YES | 1.82 (2.72,4.47) |  | 2.23 (6.81,20.00) |  | 1.08 (1.14,2.13) |  |
| ECS | NO | 1.83 (2.77,4.55) | 0.446 | 2.43 (7.24,15.33) | 0.178 | 0.80 (1.18,2.01) | 0.332 |
|  | YES | 1.90 (2.75,6.68) |  | 2.94 (12.17,39.67) |  | 1.01 (1.21,2.81) |  |
| HPVp16 | Neg | 1.57 (2.21,4.63) | 0.755 | 2.18 (3.19,31.99) | 0.700 | 1.00 (0.86,1.73) | 0.765 |
|  | Pos | 0.90 (1.12,5.33) |  | 2.20 (-6.73,30.13) |  | 0.90 (0.33,1.73) |  |
| HPVish | Neg | 1.83 (2.16,5.06) | 0.047 | 1.95 (0.84,15.76) | 0.745 | 1.26 (0.93,3.31) | 0.074 |
|  | Pos | 0.68 (-0.12,2.14) |  | 2.17 (1.47,4.49) |  | 0.60 (0.17,1.17) |  |
| LN | <18 | 2.02 (2.11,3.75) | 0.642 | 2.78 (3.10,45.75) | 0.881 | 0.86 (1.17,3.00) | 0.882 |
|  | ≥18 | 1.86 (3.32,5.16) |  | 2.78 (10.99,21.14) |  | 0.95 (1.31,2.04) |  |
| DFS | DF | 1.69 (2.40,4.22) | 0.138 | 2.22 (3.69,18.28) | 0.281 | 0.78 (1.15,2.15) | 0.248 |
|  | R/P | 1.82 (3.00,5.33) |  | 2.43 (11.82,32.94) |  | 0.99 (1.41,2.71) |  |
| Status | Alive | 1.77 (2.85,4.45) | 0.382 | 2.19 (7.15,19.40) | 0.042 | 0.80 (1.11,1.89) | 0.058 |
|  | Died | 1.78 (3.07,5.82) |  | 3.62 (12.84,29.72) |  | 1.08 (1.50,2.50) |  |

95% CI: 95%confidence interval, Gender F: Female, M: Male, T: T stage, N: N stage, TP53 W: Wild type, MU: Mutate, PNI: Perineural Invasion, ALI: Angiolymphatic Invasion, ECS: Extracapsular spread pathologic, HPV p16: HPV status (p16), HPV ish: HPV status (ish), LN: Lymph node(s) examined number, DFS: Disease Free Status, DF: Disease free, R/P: Recurred/Progressed, Status: Patient status, Neg: Negative, Pos: Positive.
