# Supplementary material for: A prognostic long non-coding RNA-associated competing endogenous RNA network in head and neck squamous cell carcinoma
Source: PeerJ. 2020 Sep 15;8:e9701. doi: 10.7717/peerj.9701 (PMC7500352; doi:10.7717/peerj.9701)
Supplement: Supplemental Information 3 [file peerj-08-9701-s003.docx]

| Clinicopathological features | | **hsa-mir-411** | | **hsa-mir-4510** | | **hsa-mir-410** | | **hsa-mir-99a** | | **hsa-mir-499a** | |
| --- | --- | --- | --- | --- | --- | --- | --- | --- | --- | --- | --- |
|  |  | median (95% CI) | p | median (95% CI) | p | median (95% CI) | p | median (95% CI) | p | median (95% CI) | p |
| Age | <65 | 33.46 (44.92,60.91) | 0.932 | 0.60 (0.80,1.16) | 0.849 | 41.36 (54.32,70.20) | 0.808 | 1304.41 (1699.28,2292.62) | 0.179 | 1.87 (3.94,9.93) | 0.009 |
|  | ≥65 | 32.63 (40.64,57.50) |  | 0.76 (0.67,1.19) |  | 38.54 (53.47,73.65) |  | 1175.72 (1394.02,1968.38) |  | 1.16 (0.32,15.56) |  |
| Gender | F | 35.01 (39.46,54.77) | 0.763 | 0.62 (0.67,1.22) | 0.831 | 46.79 (50.37,70.63) | 0.797 | 1236.29 (1290.21,1826.19) | 0.423 | 1.59 (-0.28,21.90) | 0.433 |
|  | M | 31.62 (45.49,60.51) |  | 0.63 (0.79,1.14) |  | 38.61 (55.90,71.16) |  | 1229.29 (1716.62,2263.07) |  | 1.45 (3.54,8.65) |  |
| T | T1-2 | 32.92 (41.88,61.13) | 0.151 | 0.50 (0.67,1.16) | 0.996 | 40.02 (54.95,75.29) | 0.576 | 1330.01 (1651.26,2392.02) | 0.208 | 2.11 (3.00,20.70) | 0.009 |
|  | T3-4 | 35.03 (46.74,64.48) |  | 0.61 (0.72,1.06) |  | 45.96 (57.57,75.96) |  | 1168.02 (1380.99,1823.49) |  | 1.29 (3.00,6.69) |  |
| N | N0 | 30.00 (35.46,46.19) | 0.042 | 0.03 (0.72,1.27) | 0.673 | 34.88 (50.02,71.19) | 0.141 | 1329.53 (1504.77,2134.62) | 0.223 | 1.04 (2.61,5.32) | 0.010 |
|  | N1-3 | 35.88 (52.25,75.24) |  | 0.04 (0.70,1.03) |  | 46.32 (60.11,80.11) |  | 1133.77 (1418.00,1964.77) |  | 2.03 (3.87,17.97) |  |
| Stage | I-II | 41.66 (39.05,52.40) | 0.980 | 0.50 (0.55,1.24) | 0.732 | 48.48 (51.26,71.14) | 0.849 | 1230.20 (1392.01,2069.48) | 0.613 | 1.82 (3.28,7.94) | 0.332 |
|  | III-IV | 33.84 (49.06,65.98) |  | 0.61 (0.74,1.06) |  | 45.04 (60.67,77.78) |  | 1210.83 (1517.79,2003.66) |  | 1.47 (3.49,13.63) |  |
| Grade | I-II | 32.54 (45.19,60.47) | 0.634 | 0.15 (0.72,1.06) | 0.124 | 42.74 (56.80,71.47) | 0.226 | 1204.97 (1430.13,1786.27) | 0.047 | 1.41 (2.99,11.34) | 0.183 |
|  | III-IV | 41.66 (41.22,58.41) |  | 0.89 (0.77,1.40) |  | 33.73 (47.24,73.96) |  | 1362.31 (1892.30,3229.58) |  | 1.61 (1.48,14.85) |  |
| TP53 | W | 20.04 (28.64,50.19) | <0.001 | 0.75 (0.83,1.38) | 0.137 | 26.35 (37.56,55.84) | <0.001 | 1470.10 (2087.88,3298.19) | 0.000 | 1.41 (0.24,22.58) | 0.681 |
|  | MU | 36.69 (48.81,63.09) |  | 0.61 (0.72,1.08) |  | 46.79 (61.07,76.84) |  | 1129.85 (1377.27,1752.10) |  | 1.59 (3.30,8.20) |  |
| PNI | NO | 28.80 (35.50,48.66) | <0.001 | 0.59 (0.72,1.21) | 0.898 | 34.88 (48.50,68.83) | <0.001 | 1232.87 (1564.28,2196.71) | 0.141 | 1.43 (1.52,17.43) | 0.132 |
|  | YES | 53.24 (58.59,88.35) |  | 0.73 (0.68,1.02) |  | 60.25 (66.96,93.20) |  | 1113.08 (1193.19,1640.61) |  | 1.90 (3.85,14.43) |  |
| ALI | NO | 33.95 (44.36,62.73) | 0.464 | 0.50 (0.68,1.08) | 0.216 | 40.76 (56.31,77.33) | 0.481 | 1192.60 (1419.20,1930.22) | 0.741 | 1.20 (3.82,8.01) | 0.028 |
|  | YES | 40.76 (45.92,78.87) |  | 0.74 (0.72,1.38) |  | 46.34 (57.75,86.67) |  | 1205.35 (1377.40,2093.50) |  | 2.21 (2.35,30.90) |  |
| ECS | NO | 33.50 (44.31,59.56) | 0.349 | 0.47 (0.70,1.10) | 0.373 | 38.07 (54.18,72.64) | 0.140 | 1263.59 (1405.07,1866.41) | 0.194 | 1.57 (3.40,7.07) | 0.174 |
|  | YES | 37.32 (46.81,86.04) |  | 0.76 (0.67,1.21) |  | 52.60 (57.30,89.12) |  | 907.69 (1263.28,2086.47) |  | 1.90 (1.44,34.62) |  |
| HPVp16 | Neg | 31.90 (39.59,91.60) | <0.001 | 0.14 (0.49,1.04) | 0.044 | 41.84 (49.03,83.12) | <0.001 | 1120.64 (1124.58,1903.13) | 0.001 | 1.41 (2.35,10.66) | 0.956 |
|  | Pos | 10.61 (8.92,30.37) |  | 1.31 (0.78,2.09) |  | 13.58 (-0.57,88.26) |  | 2398.75 (2261.17,4684.67) |  | 1.46 (0.05,7.68) |  |
| HPVish | Neg | 33.22 (37.70,99.36) | <0.001 | 0.52 (0.54,1.21) | 0.250 | 41.36 (46.07,82.37) | <0.001 | 1109.56 (1027.28,1936.97) | <0.001 | 1.43 (1.87,12.18) | 0.994 |
|  | Pos | 8.64 (6.42,19.18) |  | 1.00 (0.63,1.88) |  | 9.07 (7.96,18.44) |  | 3052.39 (2392.90,5450.77) |  | 1.46 (0.87,4.50) |  |
| LN | <18 | 29.91 (35.97,70.29) | 0.173 | 0.09 (0.41,1.28) | 0.156 | 39.78 (52.57,97.59) | 0.779 | 1208.16 (1331.59,2464.01) | 0.888 | 2.08 (-1.93,35.99) | 0.900 |
|  | ≥18 | 35.06 (47.71,63.04) |  | 0.71 (0.79,1.09) |  | 43.66 (57.10,71.30) |  | 1206.98 (1438.70,1863.12) |  | 1.60 (4.24,7.76) |  |
| DFS | DF | 27.27 (36.20,50.63) | 0.042 | 0.64 (0.87,1.40) | 0.637 | 32.12 (46.25,65.00) | 0.010 | 1309.17 (1872.29,2664.82) | 0.142 | 1.16 (2.63,5.96) | 0.036 |
|  | R/P | 32.98 (39.89,65.56) |  | 0.74 (0.66,1.04) |  | 40.03 (53.97,76.33) |  | 1201.93 (1285.45,1774.63) |  | 1.75 (2.29,25.33) |  |
| Status | Alive | 28.68 (38.81,54.29) | <0.001 | 0.67 (0.85,1.28) | 0.205 | 34.01 (49.95,66.51) | <0.001 | 1342.66 (1835.08,2465.18) | 0.015 | 1.19 (3.27,6.57) | 0.005 |
|  | Died | 47.57 (50.92,68.78) |  | 0.44 (0.61,0.95) |  | 56.21 (61.27,79.57) |  | 1094.12 (1210.83,1620.26) |  | 2.16 (2.61,19.96) |  |

Supplement Table 3 Relationships between the expression of miRNAs and clinicopathological features in HNSCC patients

| Clinicopathological features | | **hsa-mir-4652** | | **hsa-mir-206** | | **hsa-mir-520e** | |
| --- | --- | --- | --- | --- | --- | --- | --- |
|  |  | median (95% CI) | p | median (95% CI) | p | median (95% CI) | p |
| Age | <65 | 14.63 (17.57, 23.04) | 0.864 | 326.02 (7615.70,12300.51) | 0.136 | 0.08 (-1.24,6.73) | 0.455 |
|  | ≥65 | 12.52 (16.64,22.44) |  | 154.85 (4251.41,8597.23) |  | 0.13 (0.44,1.45) |  |
| Gender | F | 12.11 (14.08,20.33) | 0.190 | 346.51 (5435.64,11974.58) | 0.604 | 0.19 (0.41,1.94) | 0.112 |
|  | M | 14.53 (18.52,23.50) |  | 221.18 (6644.83,10573.11) |  | 0.08 (-0.91,5.54) |  |
| T | T1-2 | 11.25 (13.99,20.47) | 0.002 | 1384.10 (8779.08,15527.48) | 0.001 | 0.18 (0.56,1.63) | 0.037 |
|  | T3-4 | 16.63 (18.99,24.69) |  | 227.50 (5183.30,9305.46) |  | 0.09 (-1.56,7.54) |  |
| N | N0 | 12.47 (15.17,21.84) | 0.185 | 264.38 (6414.50,11832.17) | 0.825 | 0.10 (0.50,1.70) | 0.700 |
|  | N1-3 | 14.76 (18.00,23.56) |  | 461.21 (6870.67,12505.48) |  | 0.10 (-1.93,8.67) |  |
| Stage | I-II | 12.03 (14.39,24.08) | 0.302 | 1795.68 (8331.10,15445.57) | 0.001 | 0.90 (0.59,2.51) | 0.012 |
|  | III-IV | 14.96 (18.11,23.06) |  | 260.55 (6455.25,10884.03) |  | 0.09 (-1.10,6.06) |  |
| Grade | I-II | 13.89 (17.23,21.52) | 0.966 | 285.10 (6929.15,10762.81) | 0.246 | 0.09 (0.56,1.16) | 0.842 |
|  | III-IV | 13.70 (16.56,27.07) |  | 175.22 4732.53,12510.97) |  | 0.11 (-5.29,18.38) |  |
| TP53 | W | 10.23 (12.23,18.37) | 0.003 | 89.44 (3275.91,8673.45) | 0.001 | 0.06 (-4.31,14.96) | 0.298 |
|  | MU | 15.39 (19.20,24.22) |  | 509.22 (7438.79,11616.91) |  | 0.10 (0.61,1.25) |  |
| PNI | NO | 15.09 (18.13,25.39) | 0.416 | 76.64 (4640.70,9900.34) | <0.001 | 0.07 (0.27,1.33) | 0.018 |
|  | YES | 14.14 (15.72,22.58) |  | 2989.46 (10133.45,17720.54) |  | 0.34 (-2.60,11.88) |  |
| ALI | NO | 13.02 (15.54,20.35) | 0.112 | 238.88 (7478.43,12595.29) | 0.430 | 0.11 (0.60,1.55) | 0.742 |
|  | YES | 17.03 (19.35,30.74) |  | 976.11 (6974.64,16631.98) |  | 0.15 (0.46,0.88) |  |
| ECS | NO | 13.18 (15.90,21.13) | 0.637 | 326.02 (7110.79,12004.48) | 0.838 | 0.09 (0.48,1.30) | 0.102 |
|  | YES | 14.14 (14.97,21.96) |  | 398.97 (5713.55,15369.46) |  | 0.15 (-5.42,20.03) |  |
| HPVp16 | Neg | 15.23 (16.41,27.51) | 0.544 | 1903.09 (6220.67,16427.52) | <0.001 | 0.08 (0.39,1.41) | 0.190 |
|  | Pos | 14.19 (11.02,23.75) |  | 20.95 (-383.15,3136.98) |  | 0.04 (-0.09,0.41) |  |
| HPVish | Neg | 16.06 (16.65,29.36) | 0.891 | 1328.65 (5189.72,17111.46) | <0.001 | 0.09 (0.25,1.23) | 0.275 |
|  | Pos | 14.82 (10.27,26.47) |  | 18.97 (-1167.42,3922.54) |  | 0.05 (0.02,0.09) |  |
| LN | <18 | 13.13 (16.20,29.76) | 0.571 | 153.22 (4307.14,15772.23) | 0.248 | 0.08 (0.39,1.05) | 0.780 |
|  | ≥18 | 13.71 (16.58,20.94) |  | 412.42 (7525.67,11576.77) |  | 0.10 (-1.03,6.33) |  |
| DFS | DF | 12.47 (15.21,20.00) | 0.337 | 147.04 (5071.20,8665.49) | 0.047 | 0.08 (0.51,0.99) | 0.728 |
|  | R/P | 13.48 (16.02,23.19) |  | 366.31 (6853.70,14935.89) |  | 0.10 (0.44,1.04) |  |
| Status | Alive | 12.54 (15.71,19.86) | 0.025 | 165.74 (5969.64,9770.09) | 0.060 | 0.08 (0.49,0.88) | 0.183 |
|  | Died | 15.73 (19.69,27.88) |  | 437.41 (6739.21,13125.30) |  | 0.12 (-2.06,10.33) |  |

95% CI: 95%confidence interval, Gender F: Female, M: Male, T: T stage, N: N stage, TP53 W: Wild type, MU: Mutate, PNI: Perineural Invasion, ALI: Angiolymphatic Invasion, ECS: Extracapsular spread pathologic, HPV p16: HPV status (p16), HPV ish: HPV status (ish), LN: Lymph node(s) examined number, DFS: Disease Free Status, DF: Disease free, R/P: Recurred/Progressed, Status: Patient status, Neg: Negative, Pos: Positive.
