# Supplementary material for: A prognostic long non-coding RNA-associated competing endogenous RNA network in head and neck squamous cell carcinoma
Source: PeerJ. 2020 Sep 15;8:e9701. doi: 10.7717/peerj.9701 (PMC7500352; doi:10.7717/peerj.9701)
Supplement: Supplemental Information 4 [file peerj-08-9701-s004.docx]

| Clinicopathological features | | **CELSR3** | | **STC2** | | **ADGRD2** | | **ZNF541** | | **GRB14** | |
| --- | --- | --- | --- | --- | --- | --- | --- | --- | --- | --- | --- |
|  |  | median (95% CI) | p | median (95% CI) | p | median (95% CI) | p | median (95% CI) | p | median (95% CI) | p |
| Age | <65 | 424.31 (673.96,864.46) | 0.255 | 961.67 (1281.13,1604.17) | 0.898 | 27.15 (43.07,58.33) | 0.410 | 11.45 (141.86,233.32) | 0.009 | 31.26 (56.49,91.37) | 0.256 |
|  | ≥65 | 379.00 (557.89,773.01) |  | 911.24 (1340.16,2211.77) |  | 28.44 (38.52,65.80) |  | 9.05 (53.09,130.30) |  | 24.05 (44.57,69.05) |  |
| Gender | F | 372.46 (556.43,824.22) | 0.547 | 866.40 (1113.38,1538.30) | 0.421 | 18.00 (32.34,60.35) | 0.021 | 8.90 (28.70,91.25) | 0.035 | 21.93 (38.91,68.66) | 0.162 |
|  | M | 412.07 (659.53,830.12) |  | 970.86 (1404.12,1895.93) |  | 30.69 (44.93,60.97) |  | 11.35 (142.37,225.87) |  | 31.87 (57.26,87.60) |  |
| T | T1-2 | 418.62 (608.54,831.09) | 0.151 | 707.43 (1035.87,1528.26) | <0.001 | 23.70 (35.64,59.24) | 0.072 | 11.24 (116.27,237.01) | 0.020 | 26.12 (42.41,94.98) | 0.685 |
|  | T3-4 | 342.59 (629.25,842.09) |  | 1146.03 (1428.17,1961.66) |  | 31.55 (43.13,58.60) |  | 8.78 (52.63,115.60) |  | 28.31 (54.28,80.22) |  |
| N | N0 | 408.38 (614.62,858.83) | 0.413 | 872.66 (1167.14,1622.40) | 0.157 | 32.36 (45.47,69.70) | 0.018 | 8.95 (56.55,141.40) | 0.488 | 28.54 (46.55,76.46) | 0.861 |
|  | N1-3 | 351.43 (619.39,845.24) |  | 1070.59 (1308.11,1877.17) |  | 25.34 (36.72,53.06) |  | 9.71 (87.19,173.82) |  | 28.07 (51.10,94.01) |  |
| Stage | I-II | 409.85 (535.50,800.45) | 0.618 | 676.81 (950.66,1702.91) | 0.003 | 21.56 (30.24,65.23) | 0.271 | 9.65 (51.61,178.83) | 0.684 | 23.70 (37.76,71.16) | 0.398 |
|  | III-IV | 355.28 (654.30,844.28) |  | 1054.18 (1402.79,1875.31) |  | 30.60 (42.03,55.71) |  | 9.09 (82.05,151.66) |  | 27.89 (55.12,88.41) |  |
| Grade | I-II | 374.05 (610.69,770.55) | 0.161 | 946.32 (1288.78,1616.98) | 0.872 | 28.17 (41.71,57.89) | 0.424 | 9.09 (68.75,130.97) | 0.003 | 27.85 (51.91,72.55) | 0.658 |
|  | III-IV | 425.23 (642.66,959.67) |  | 912.77 (1275.73,2478.88) |  | 26.23 (38.85,67.38) |  | 14.31 (167.47,326.42) |  | 34.77 (45.78,122.65) |  |
| TP53 | W | 823.48 (921.51,1275.87) | <0.001 | 752.82 (947.97,1343.13) | 0.001 | 36.85 (54.88,87.63) | 0.001 | 30.01 (336.30,528.62) | <0.001 | 21.38 (39.55,74.63) | 0.004 |
|  | MU | 318.00 (522.03,661.17) |  | 1042.73 (1481.15,1986.70) |  | 24.34 (36.32,51.01) |  | 8.97 (31.03,64.91) |  | 30.91 (56.13,86.45) |  |
| PNI | NO | 459.57 (655.71,909.39) | 0.260 | 907.76 (1220.94,1825.91) | 0.020 | 31.08 (42.36,65.26) | 0.367 | 11.45 (113.37,225.53) | 0.001 | 27.42 (50.79,79.02) | 0.138 |
|  | YES | 369.86 (591.40,839.16) |  | 1071.12 (1370.03,2180.53) |  | 26.21 (37.63,58.75) |  | 7.51 (28.23,97.80) |  | 35.07 (55.21,115.83) |  |
| ALI | NO | 402.50 (649.46,868.77) | 0.518 | 919.64 (1260.29,1736.40) | 0.237 | 28.64 (42.99,63.25) | 0.567 | 9.71 (69.80,156.39) | 0.379 | 28.77 (55.47,84.06) | 0.874 |
|  | YES | 415.30 (514.97,752.26) |  | 1113.26 (1224.68,2318.74) |  | 26.95 (34.00,52.65) |  | 9.19 (92.14,237.75) |  | 34.77 (38.86,118.07) |  |
| ECS | NO | 406.91 (605.17,801.28) | 0.391 | 881.74 (1189.24,1658.56) | 0.004 | 28.92 (38.87,53.79) | 0.738 | 9.56 (52.85,122.15) | 0.412 | 26.06 (46.01,69.16) | 0.024 |
|  | YES | 319.16 (530.38,876.02) |  | 1274.85 (1444.93,2101.49) |  | 29.08 (33.26,57.72) |  | 9.72 (78.88,228.67) |  | 37.65 (57.29,145.25) |  |
| HPVp16 | Neg | 376.85 (438.21,699.82) | <0.001 | 1113.26 (1164.38,2309.55) | 0.194 | 15.60 (27.73,52.07) | <0.001 | 9.99 (22.63,72.44) | <0.001 | 28.54 (51.64,115.44) | 0.459 |
|  | Pos | 1047.66 (937.74,1916.44) |  | 752.82 (783.71,1675.88) |  | 65.99 (60.43,150.17) |  | 878.12 (655.17,1050.15) |  | 31.85 (27.08,95.17) |  |
| HPVish | Neg | 329.76 (429.38,773.28) | <0.001 | 1402.14 (1341.86,2194.58) | 0.024 | 18.17 (27.31,55.09) | <0.001 | 9.88 (22.57,71.90) | <0.001 | 32.53 (44.95,118.30) | 0.301 |
|  | Pos | 1071.30 (992.23,2205.24) |  | 664.55 (497.18,1623.74) |  | 77.52 (58.44,193.23) |  | 954.19 (824.50,1274.57) |  | 33.53 (17.33,76.03) |  |
| LN | <18 | 389.57 (484.17,808.10) | 0.846 | 893.98 (1008.32,2388.87) | 0.498 | 24.67 (29.20,48.10) | 0.428 | 11.90 (31.35,133.26) | 0.219 | 37.08 (31.84,141.00) | 0.380 |
|  | ≥18 | 374.05 (622.47,791.50) |  | 1042.73 (1362.27,1777.71) |  | 28.44 (41.79,59.75) |  | 9.00 (76.54,144.51) |  | 27.89 (53.96,75.78) |  |
| DFS | DF | 537.60 (690.96,893.44) | 0.121 | 774.91 (1051.78,1330.75) | 0.032 | 34.22 (50.30,71.60) | 0.061 | 12.54 (166.43,276.32) | <0.001 | 22.17 (41.80,62.70) | 0.040 |
|  | R/P | 403.26 (624.17,924.12) |  | 998.75 (1419.61,2227.04) |  | 25.92 (35.62,52.89) |  | 8.78 (51.11,160.91) |  | 33.41 (51.80,119.95) |  |
| Status | Alive | 506.60 (697.38,880.01) | 0.001 | 820.58 (1177.05,1518.91) | 0.002 | 32.09 (49.46,67.22) | <0.001 | 12.22 (153.33,247.31) | <0.001 | 23.86 (46.49,66.96) | 0.003 |
|  | Died | 312.54 (517.01,749.53) |  | 1202.11 (1513.64,2352.94) |  | 19.21 (28.30,50.31) |  | 8.03 (38.04,103.62) |  | 37.05 (59.07,112.42) |  |

Supplement Table 4 Relationships between the expression of mRNAs and clinicopathological features in HNSCC patients

| Clinicopathological features | | **NOSTRIN** | | **TIMP4** | | **HOXB9** | | **ADPRHL1** | | **SPINK1** | |
| --- | --- | --- | --- | --- | --- | --- | --- | --- | --- | --- | --- |
|  |  | median (95% CI) | p | median (95% CI) | p | median (95% CI) | p | median (95% CI) | p | median (95% CI) | p |
| Age | <65 | 88.20 (94.93,105.96) | 0.205 | 15.75 (25.26,34.76) | 0.623 | 45.93 (104.13,181.07) | 0.191 | 126.11 (177.66,255.69) | 0.059 | 6.66 (20.18,36.94) | 0.987 |
|  | ≥65 | 86.24 (88.16,104.53) |  | 15.23 (21.09,34.95) |  | 52.54 (102.07,244.24) |  | 150.92 (169.55,230.39) |  | 6.82 (23.02,63.28) |  |
| Gender | F | 80.91 (87.33,107.73) | 0.179 | 15.41 (20.06,31.59) | 0.905 | 41.80 (74.84,240.63) | 0.829 | 128.16 (168.13,293.12) | 0.625 | 6.55 (13.04,53.88) | 0.769 |
|  | M | 89.89 (94.30,104.52) |  | 15.73 (25.56,35.39) |  | 53.31 (113.87,191.62) |  | 132.25 (174.20,232.73) |  | 6.77 (24.05,44.23) |  |
| T | T1-2 | 89.89 (97.50,114.74) | 0.014 | 17.23 (22.91,34.86) | 0.508 | 34.09 (73.84,225.76) | 0.020 | 148.64 (178.50,261.80) | 0.326 | 5.59 (14.87,33.84) | 0.006 |
|  | T3-4 | 84.15 (87.05,98.26) |  | 16.21 (25.32,36.79) |  | 64.13 (110.02,190.56) |  | 128.85 (172.87,254.76) |  | 8.10 (27.24,58.48) |  |
| N | N0 | 86.39 (92.14,108.89) | 0.951 | 16.02 (20.54,30.34) | 0.061 | 39.39 (62.39,127.96) | 0.087 | 115.36 (159.52,222.90) | 0.506 | 8.45 (25.24,61.31) | 0.310 |
|  | N1-3 | 89.52 (91.51,103.22) |  | 19.01 (27.28,40.85) |  | 61.58 (126.15,248.94) |  | 137.13 (181.77,267.86) |  | 6.67 (19.16,46.37) |  |
| Stage | I-II | 88.12 (92.59,118.35) | 0.290 | 16.56 (17.52,27.10) | 0.151 | 36.40 (29.66,187.56) | 0.059 | 135.38 (160.19,233.24) | 0.519 | 5.84 (9.74,34.70) | 0.145 |
|  | III-IV | 86.24 (90.38,100.68) |  | 17.78 (27.00,37.42) |  | 56.62 (116.52,208.18) |  | 133.88 (185.20,260.04) |  | 7.04 (26.46,52.23) |  |
| Grade | I-II | 85.82 (91.35,101.38) | 0.105 | 15.00 (23.64,33.08) | 0.151 | 41.97 (82.93,157.47) | 0.068 | 135.53 (177.65,231.48) | 0.580 | 6.77 (26.05,49.81) | 0.116 |
|  | III-IV | 94.38 (96.13,119.26) |  | 21.39 (25.91,42.10) |  | 69.63 (138.97,317.55) |  | 123.57 (155.58,273.53) |  | 6.33 (10.29,29.77) |  |
| TP53 | W | 97.23 (98.18,119.92) | 0.032 | 9.31 (15.27,35.17) | <0.001 | 38.15 (72.98,239.36) | 0.123 | 92.51 (137.85,220.70) | 0.001 | 4.44 (9.44,37.11) | <0.001 |
|  | MU | 85.47 (90.19,99.86) |  | 18.89 (26.88,34.97) |  | 55.24 (114.08,191.77) |  | 142.30 (187.99,256.23) |  | 8.32 (26.70,49.70) |  |
| PNI | NO | 91.80 (95.48,109.60) | 0.016 | 17.26 (26.03,39.81) | 0.442 | 36.40 (79.62,212.36) | 0.354 | 124.08 (159.56,254.92) | 0.002 | 6.38 (17.94,39.32) | 0.846 |
|  | YES | 83.96 (85.11,101.01) |  | 21.00 (25.01,40.83) |  | 55.24 (98.81,209.02) |  | 171.35 (213.71,329.26) |  | 7.04 (17.94,39.32) |  |
| ALI | NO | 87.93 (90.17,102.65) | 0.923 | 16.99 (23.33,36.83) | 0.138 | 36.77 (76.05,184.71) | 0.009 | 134.07 (181.60,249.80) | 0.340 | 6.38 (18.92,41.43) | 0.399 |
|  | YES | 84.61 (88.79,107.17) |  | 20.09 (26.98,44.17) |  | 70.80 (126.64,330.25) |  | 168.88 (184.65,333.25) |  | 7.64 (14.49,38.05) |  |
| ECS | NO | 86.75 (91.95,104.11) | 0.568 | 18.66 (24.92,35.45) | 0.104 | 36.77 (78.59,147.47) | 0.001 | 136.17 (177.29,236.54) | 0.408 | 6.31 (19.32,44.50) | 0.208 |
|  | YES | 87.11 (86.00,103.62) |  | 22.27 (28.26,53.30) |  | 86.97 (130.06,367.53) |  | 150.98 (188.96,387.71) |  | 8.15 (23.14,62.09) |  |
| HPVp16 | Neg | 90.28 (94.73,118.86) | 0.234 | 27.92 (27.90,44.39) | <0.001 | 39.27 (65.08,181.94) | 0.009 | 153.50 (161.48,358.03) | <0.001 | 6.48 (6.26,40.31) | 0.976 |
|  | Pos | 103.27 (98.70,139.04) |  | 8.30 (7.40,33.06) |  | 124.12 (90.28,637.67) |  | 67.87 (59.06,139.18) |  | 8.01 (-8.60,86.21) |  |
| HPVish | Neg | 86.98 (89.90,115.48) | 0.150 | 28.03 (26.88,46.29) | <0.001 | 61.52 (66.52,194.27) | 0.234 | 162.18 (134.00,353.39) | 0.001 | 6.85 (3.95,46.36) | 0.051 |
|  | Pos | 103.93 (95.44,135.82) |  | 6.20 (4.45,9.85) |  | 118.66 (66.89,392.34) |  | 52.48 (44.62,127.78) |  | 3.99 (2.66,8.48) |  |
| LN | <18 | 85.46 (88.17,106.41) | 0.678 | 15.41 (22.21,44.12) | 0.480 | 46.48 (78.79,407.65) | 0.796 | 142.30 (149.92,302.66) | 0.890 | 6.48 (14.27,31.71) | 0.718 |
|  | ≥18 | 87.28 (91.96,102.63) |  | 18.66 (26.12,36.24) |  | 45.93 (100.91,151.85) |  | 131.52 (181.96,242.08) |  | 6.68 (25.52,51.65) |  |
| DFS | DF | 100.82 (100.98,115.33) | 0.004 | 11.66 (19.26,28.71) | 0.004 | 35.89 (97.82,207.56) | 0.080 | 111.17 (144.72,194.69) | 0.003 | 6.08 (16.10,35.73) | 0.245 |
|  | R/P | 83.06 (85.98,100.62) |  | 21.13 (26.25,40.45) |  | 49.88 (77.98,225.77) |  | 152.85 (190.97,300.89) |  | 6.93 (20.81,59.36) |  |
| Status | Alive | 97.03 (100.35,112.61) | <0.001 | 13.48 (21.60,29.76) | 0.015 | 39.94 (109.05,198.39) | 0.065 | 123.01 (155.77,210.37) | 0.009 | 6.48 (19.80,41.90) | 0.273 |
|  | Died | 75.16 (79.83,92.61) |  | 19.49 (27.30,43.23) |  | 65.27 (94.47,214.63) |  | 150.72 (201.01,312.15) |  | 7.69 (23.23,55.19) |  |

| Clinicopathological features | | **PTX3** | | **FRZB** | | **ODF4** | | **PLAU** | | **OLR1** | |
| --- | --- | --- | --- | --- | --- | --- | --- | --- | --- | --- | --- |
|  |  | median (95% CI) | p | median (95% CI) | p | median (95% CI) | p | median (95% CI) | p | median (95% CI) | p |
| Age | <65 | 55.67 (146.99,245.88) | 0.269 | 115.91 (191.42,276.38) | 0.075 | 1.04 (1.73,3.41) | 0.871 | 14153.90 (15831.45,18880.56) | 0.989 | 189.88 (371.80,561.23) | 0.115 |
|  | ≥65 | 46.95 (96.38,248.99) |  | 89.45 (128.73,174.70) |  | 1.04 (1.64,2.69) |  | 13924.65 (15821.69,20636.61) |  | 237.08 (472.61,881.59) |  |
| Gender | F | 53.93 (105.95,261.37) | 0.516 | 112.88 (152.55,255.75) | 0.550 | 1.04 (1.93,3.61) | 0.486 | 15658.80 (16380.41,22105.70) | 0.173 | 267.06 (464.17,897.79) | 0.014 |
|  | M | 51.61 (139.21,238.82) |  | 100.99 169.50,236.85) |  | 1.04 (1.59,2.97) |  | 13929.42 (15679.49,18590.12) |  | 179.42 (390.98,603.12) |  |
| T | T1-2 | 50.35 (95.54,221.19) | 0.344 | 115.46 (177.09,287.73) | 0.037 | 1.17 (2.12,3.45) | 0.147 | 13977.65 (14841.85,19545.98) | 0.062 | 196.37 (376.76,682.48) | 0.684 |
|  | T3-4 | 55.29 (150.79,280.84) |  | 98.84 131.86,190.32) |  | 1.02 (1.36,3.23) |  | 15174.32 (17003.47,20468.69) |  | 218.51 (444.69,736.37) |  |
| N | N0 | 52.20 (115.17,274.58) | 0.964 | 91.98 (134.74,229.24) | 0.124 | 1.06 (1.82,3.06) | 0.725 | 13238.30 (15067.67,20024.96) | 0.110 | 170.12 (397.90,844.66) | 0.145 |
|  | N1-3 | 53.43 (134.37,238.69) |  | 109.48 (154.79,234.31) |  | 1.09 (1.53,3.62) |  | 15556.16 (16770.80,20417.55) |  | 232.90 (426.93,659.92) |  |
| Stage | I-II | 50.15 (76.45,166.73) | 0.354 | 105.57 (145.39,271.43) | 0.675 | 1.40 (2.13,4.08) | 0.134 | 13965.61 (14326.81,21334.88) | 0.236 | 230.52 (337.54,858.35) | 0.630 |
|  | III-IV | 55.37 (147.62,253.21) |  | 99.21 (145.94,199.81) |  | 1.07 (1.56,3.07) |  | 15221.97 (17024.09,20118.70) |  | 216.36 (447.07,686.07) |  |
| Grade | I-II | 48.96 (107.46,175.15) | 0.166 | 97.25 (145.68,198.03) | 0.005 | 1.09 (1.83,2.59) | 0.193 | 14396.95 (16374.91,19425.22) | 0.475 | 199.25 (404.49,622.57) | 0.667 |
|  | III-IV | 67.47 (186.21,460.07) |  | 121.46 (211.91,378.36) |  | 0.87 (1.20,5.38) |  | 13027.53 (14938.55,20691.17) |  | 223.25 (441.59,904.18) |  |
| TP53 | W | 32.64 (101.04,260.57) | 0.003 | 143.55 (223.77,379.95) | 0.001 | 1.27 (1.89,3.31) | 0.068 | 8619.30 (11439.47,16226.26) | <0.001 | 170.12 (267.24,589.54) | 0.017 |
|  | MU | 60.81 (137.07,235.03) |  | 96.68 (140.88,191.52) |  | 0.95 (1.59,3.05) |  | 15987.10 (17583.22,20696.99) |  | 219.00 (473.45,713.32) |  |
| PNI | NO | 46.80 (104.44,279.80) | 0.014 | 106.91 (159.92,267.49) | 0.419 | 1.16 (1.56,4.13) | 0.077 | 11137.36 (12286.84,16152.35) | <0.001 | 162.39 (363.28,757.26) | 0.013 |
|  | YES | 64.41 (135.95,273.08) |  | 100.29 (134.25,209.12) |  | 0.95 (1.21,2.13) |  | 20696.66 (20383.65,24766.94) |  | 255.82 (492.46,823.93) |  |
| ALI | NO | 52.20 (130.10,287.46) | 0.835 | 98.94 (146.18,219.27) | 0.386 | 0.06 (1.67,2.65) | 0.959 | 15012.94 (15902.94,19926.79) | 0.425 | 199.25 (457.62,820.00) | 0.718 |
|  | YES | 55.67 (94.10,171.25) |  | 116.17 (142.17,289.03) |  | 0.07 (0.63,4.85) |  | 15516.87 (16032.69,20935.92) |  | 208.99 (355.33,688.15) |  |
| ECS | NO | 52.42 (119.45,261.39) | 0.262 | 101.23 (154.50,246.33) | 0.708 | 1.05 (1.85,3.86) | 0.112 | 14380.13 (15983.57,19564.58) | 0.167 | 190.86 (404.82,734.47) | 0.025 |
|  | YES | 63.56 (128.91,318.21) |  | 105.52 (124.72,205.14) |  | 0.93 (1.07,2.18) |  | 17355.47 (16693.06,21905.65) |  | 280.58 (505.16,925.98) |  |
| HPVp16 | Neg | 54.43 (77.59,170.35) | 0.090 | 125.11 (150.40,264.79) | 0.013 | 1.40 (0.62,7.14) | 0.825 | 15090.94 (13917.51,19129.04) | <0.001 | 219.37 (322.39,724.62) | 0.001 |
|  | Pos | 27.99 (46.34,341.94) |  | 221.56 (191.00,674.27) |  | 1.31 (1.06,3.63) |  | 4720.65 (4152.67,9364.45) |  | 73.14 (79.77,197.32) |  |
| HPVish | Neg | 46.88 (65.10,400.70) | 0.047 | 126.07 (131.74,234.80) | 0.006 | 1.22 (1.41,3.06) | 0.352 | 15282.69 (13793.38,19409.36) | <0.001 | 236.03 (379.64,935.65) | 0.005 |
|  | Pos | 18.80 (10.26,186.78) |  | 252.00 (175.55,949.48) |  | 1.87 (1.26,2.98) |  | 3980.70 (2760.65,10557.09) |  | 74.96 (64.36,184.19) |  |
| LN | <18 | 53.33 (112.61,367.50) | 0.755 | 94.60 (119.09,265.53) | 0.644 | 0.81 (1.26,2.94) | 0.757 | 14039.52 (14251.24,20713.00) | 0.411 | 195.98 (212.37,311.47) | 0.165 |
|  | ≥18 | 57.38 (132.82,224.36) |  | 102.95 (154.45,221.99) |  | 1.05 (1.73,3.29) |  | 15126.68 (17004.50,20356.96) |  | 219.00 (496.25,764.23) |  |
| DFS | DF | 41.05 (106.13,214.51) | 0.258 | 120.68 (196.63,294.89) | 0.009 | 1.17 (1.90,3.94) | 0.489 | 12029.05 (13582.61,17209.02) | 0.002 | 162.19 (325.75,564.27) | 0.002 |
|  | R/P | 56.98 (102.02,272.14) |  | 93.96 (129.01,210.83) |  | 1.06 (1.48,2.68) |  | 16055.12 (17402.84,22628.81) |  | 259.53 (421.12,846.48) |  |
| Status | Alive | 45.97 (117.84,205.52) | 0.038 | 115.69 (189.43,268.84) | 0.004 | 1.19 (2.02,3.64) | 0.003 | 12639.00 (14713.57,17981.47) | 0.001 | 162.19 (364.48,615.12) | <0.001 |
|  | Died | 62.31 (146.23,316.33) |  | 85.74 (125.05,195.34) |  | 0.78 (1.17,2.12) |  | 17297.53 (17770.41,22070.14) |  | 295.55 (486.32,787.17) |  |

| Clinicopathological features | | **GNG7** | | **DTHD1** | |
| --- | --- | --- | --- | --- | --- |
|  |  | median (95% CI) | p | median (95% CI) | p |
| Age | <65 | 94.94 (127.53,168.59) | 0.282 | 15.95 (29.21,45.38) | 0.892 |
|  | ≥65 | 84.48 (106.80,145.88) |  | 15.79 (22.91,32.30) |  |
| Gender | F | 92.04 (106.50,145.00) | 0.906 | 17.22 (19.36,45.68) | 0.977 |
|  | M | 88.72 (126.16,163.79) |  | 15.47 (28.44,39.80) |  |
| T | T1-2 | 97.75 (124.99,166.88) | 0.055 | 16.59 (26.51,49.10) | 0.406 |
|  | T3-4 | 81.37 (105.31,148.84) |  | 15.11 (25.04,37.50) |  |
| N | N0 | 83.87 (108.57,168.32) | 0.482 | 15.90 (22.42,44.61) | 0.449 |
|  | N1-3 | 93.87 (119.05,157.76) |  | 15.48 (27.19,42.17) |  |
| Stage | I-II | 83.18 (103.83,154.33) | 0.727 | 15.45 (18.56,53.62) | 0.863 |
|  | III-IV | 87.62 (116.70,155.06) |  | 15.47 (26.99,37.94) |  |
| Grade | I-II | 88.13 (109.27,131.87) | 0.412 | 15.96 (27.96,41.83) | 0.568 |
|  | III-IV | 88.69 (131.04,179.00) |  | 15.93 (22.52,38.98) |  |
| TP53 | W | 93.25 (122.99,152.24) | 0.201 | 25.73 (30.22,44.83) | <0.001 |
|  | MU | 84.08 (110.42,144.67) |  | 14.07 (25.57,39.52) |  |
| PNI | NO | 100.99 (123.94,184.33) | 0.083 | 15.99 (23.28,44.32) | 0.504 |
|  | YES | 79.84 (98.83,134.14) |  | 14.43 (22.62,38.64) |  |
| ALI | NO | 89.21 (113.38,159.61) | 0.680 | 15.47 (22.49,40.91) | 0.692 |
|  | YES | 95.35 (113.92,168.66) |  | 13.93 (23.92,43.96) |  |
| ECS | NO | 88.00 (115.31,163.19) | 0.923 | 16.04 (27.40,41.31) | 0.008 |
|  | YES | 87.71 (103.03,153.84) |  | 11.41 (15.04,26.72) |  |
| HPVp16 | Neg | 110.90 (118.36,182.66) | 0.278 | 13.49 (26.02,51.91) | 0.115 |
|  | Pos | 138.50 (142.42,302.31) |  | 23.87 (23.24,88.75) |  |
| HPVish | Neg | 105.47 (114.88,190.16) | 0.109 | 13.63 (24.74,54.70) | 0.053 |
|  | Pos | 200.22 (141.91,304.63) |  | 35.23 (22.72,79.57) |  |
| LN | <18 | 95.30 (109.13,228.77) | 0.811 | 15.72 (19.19,33.63) | 0.842 |
|  | ≥18 | 90.13 (116.55,146.03) |  | 14.98 (26.47,40.89) |  |
| DFS | DF | 98.60 (130.79,180.38) | 0.079 | 18.49 (29.37,47.44) | 0.056 |
|  | R/P | 84.39 (110.43,157.03) |  | 15.11 (22.24,39.20) |  |
| Status | Alive | 98.10 (134.01,176.00) | 0.001 | 16.93 (31.75,47.82) | 0.009 |
|  | Died | 78.51 (97.04,132.52) |  | 14.43 (18.69,28.26) |  |

95% CI: 95%confidence interval, Gender F: Female, M: Male, T: T stage, N: N stage, TP53 W: Wild type, MU: Mutate, PNI: Perineural Invasion, ALI: Angiolymphatic Invasion, ECS: Extracapsular spread pathologic, HPV p16: HPV status (p16), HPV ish: HPV status (ish), LN: Lymph node(s) examined number, DFS: Disease Free Status, DF: Disease free, R/P: Recurred/Progressed, Status: Patient status, Neg: Negative, Pos: Positive.
