# Supplementary figures and images for: A prognostic long non-coding RNA-associated competing endogenous RNA network in head and neck squamous cell carcinoma
Source: PeerJ. 2020 Sep 15;8:e9701. doi: 10.7717/peerj.9701 (PMC7500352; doi:10.7717/peerj.9701)

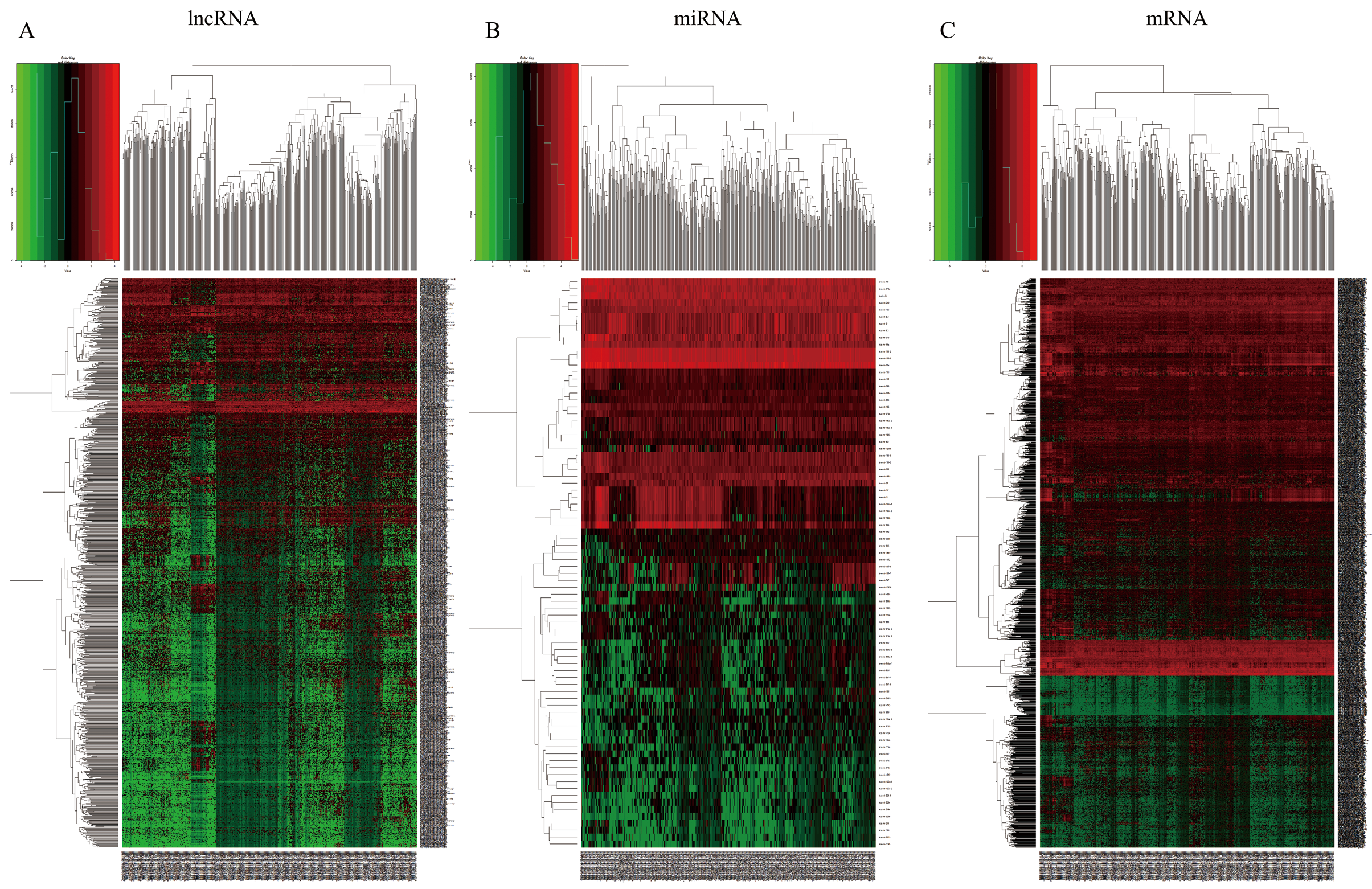

Supplement: Supplemental Information 5 — The red points represent up-regulated RNAs, green represent down-regulated RNAs, and black represent no significant difference. [file peerj-08-9701-s005.png]
